# Distinct Glycosylation Responses to Spinal Cord Injury in Regenerative and Nonregenerative Models

**DOI:** 10.1021/acs.jproteome.2c00043

**Published:** 2022-05-04

**Authors:** Rachel Ronan, Aniket Kshirsagar, Ana Lúcia Rebelo, Abbah Sunny, Michelle Kilcoyne, Roisin O’ Flaherty, Pauline M. Rudd, Gerhard Schlosser, Radka Saldova, Abhay Pandit, Siobhan S. McMahon

**Affiliations:** † SFI Research Centre for Medical Devices (CÚRAM), National University of Ireland, Galway, Galway H91 W2TY, Ireland; ‡ Discipline of Microbiology, National University of Ireland, Galway, Galway H91 W2TY, Ireland; § Department of Chemistry, Maynooth University, Maynooth, Co., Kildare W23 F2H6, Ireland; ∥ The National Institute for Bioprocessing, Research, and Training (NIBRT), Dublin A94 X099, Ireland; ⊥ Conway Institute, University College Dublin, Belfield, Dublin 4 D04 PR94, Ireland; # School of Natural Science, National University of Ireland, Galway, Galway H91 W2TY, Ireland; ∇ UCD School of Medicine, College of Health and Agricultural Science (CHAS), University College Dublin (UCD), Dublin D04 PR94, Ireland; ○ Discipline of Anatomy, National University of Ireland, Galway H91 W5P7, Ireland

**Keywords:** spinal cord injury, glycosylation, Xenopus laevis, rat, regeneration, collagen hydrogel

## Abstract

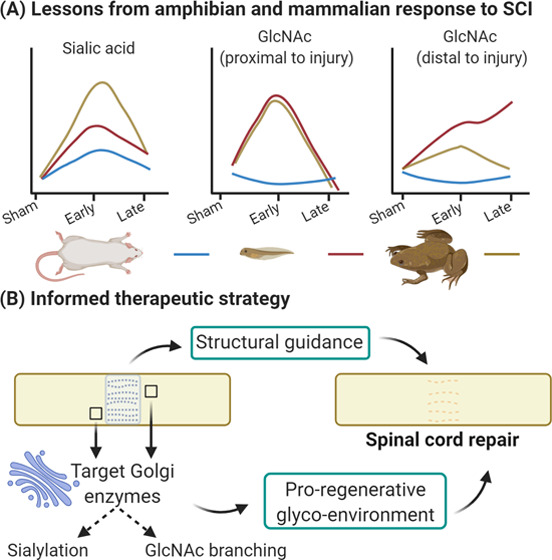

Traumatic spinal cord injury (SCI) results in disruption of tissue integrity and loss of function. We hypothesize that glycosylation has a role in determining the occurrence of regeneration and that biomaterial treatment can influence this glycosylation response. We investigated the glycosylation response to spinal cord transection in *Xenopus laevis* and rat. Transected rats received an aligned collagen hydrogel. The response compared regenerative success, regenerative failure, and treatment in an established nonregenerative mammalian system. In a healthy rat spinal cord, ultraperformance liquid chromatography (UPLC) N-glycoprofiling identified complex, hybrid, and oligomannose N-glycans. Following rat SCI, complex and outer-arm fucosylated glycans decreased while oligomannose and hybrid structures increased. Sialic acid was associated with microglia/macrophages following SCI. Treatment with aligned collagen hydrogel had a minimal effect on the glycosylation response. In *Xenopus*, lectin histochemistry revealed increased levels of *N*-acetyl-glucosamine (GlcNAc) in premetamorphic animals. The addition of GlcNAc is required for processing complex-type glycans and is a necessary foundation for additional branching. A large increase in sialic acid was observed in nonregenerative animals. This work suggests that glycosylation may influence regenerative success. In particular, loss of complex glycans in rat spinal cord may contribute to regeneration failure. Targeting the glycosylation response may be a promising strategy for future therapies.

## Introduction

The World Health Organization (WHO) estimates that between 250,000 and 500,000 new patients suffer a spinal cord injury (SCI) each year (WHO factsheet, SCI, 2013). Following SCI, motor and sensory function may be impaired below the level of the injury. Therefore, cervical and thoracic injuries tend to cause the most significant detriment to the patient.

The pathophysiology of SCI is reasonably well understood. Cellular changes include the death of neurons and oligodendrocytes, scar formation, activation of astrocytes and microglia, and the influx of peripheral macrophages.^1−4^ Wide-scale alterations in protein expression and intracellular signaling are also evident.^5−7^ However, nothing is known so far regarding the impact of SCI on glycosylation, one of the most important post-translational modifications.^8^

Glycosylation, particularly N-glycosylation, is considered an essential regulator of biological activity and influences the subcellular localization of proteins, as well as their stability, secretion, and interaction with ligands or receptors.^9^ Glycans can be essential mediators of cell–cell or cell–matrix interaction,^9^ and so understanding their expression and behavior can give a more in-depth insight into the pathogenesis and cellular behavior underlying injury and disease. Indeed, N-glycans have been recognized as having an essential role in cancer^10^ and inflammation.^11,12^ In the CNS, not much is known about their role; however, the neurological symptoms associated with the congenital disorders of glycosylation, such as seizures, motor dysfunction, or sensory impairment,^13,14^ highlight the importance of this class of molecules. A small number of studies have investigated glycosylation changes in the context of traumatic CNS injury. Changes in the abundance of a variety of N-glycans were found following cortical impact^15^ and SCI contusion models^16,17^ in the rat. Alterations in N-glycosylation have also been seen in some neurodegenerative diseases such as Alzheimer’s disease,^18−20^ Huntington’s disease,^21^ and multiple sclerosis.^22−24^ However, it is difficult to say whether these are a cause or consequence of the disease.

Interest in using biomaterials to treat SCI has been growing in recent years. Biomaterials, and collagen in particular, show promise in SCI preclinical models when used alone or in combination with other therapeutic factors,^25,26^ and a collagen scaffold combination treatment with human mesenchymal stem cells has even been brought to clinical trial.^27−29^ However, there is still vast room for improvement in their efficacy. Biomaterial systems can specifically be employed to interfere with the secondary injury events which follow SCI.^30^ A previous study by our group demonstrated that collagen hydrogel reduced the amount of ionized calcium-binding adaptor molecule 1 (Iba-1) and neuron-glial antigen 2 (NG2) chondroitin sulfate proteoglycan (CSPG)-positive staining and led to improved motor function following SCI.^31^ Considering these observed effects, we asked whether the glycosylation profile of the tissue may also be influenced by treatment with a biomaterial. From recent *in vitro* studies, it is clear that coupling glycans and collagen can influence populations of neural cells: either by driving neuronal differentiation of immortalized cell lines^32^ or by influencing the glycosylation status of primary neural cells,^33^ and this strategy will warrant investigation in SCI.

In this study, we use a collagen hydrogel for the treatment of SCI in the rat. Although collagen is a minor constituent of the CNS extracellular matrix,^34^ it is well tolerated, not eliciting any obvious foreign body response,^31,35,36^ and has been approved by FDA for peripheral nerve treatments.^37^ Being a fibrillar protein, collagen can be manipulated to provide aligned surface guidance for migrating cells or growing axons.^38−44^ Fibers were aligned in the collagen hydrogel used in this study, and this particular hydrogel has been shown to positively influence the outcome following SCI, in particular by promoting axonal outgrowth, limiting the formation of the astroglial scar, and altering the activity of numerous biological pathways. Following the establishment of the normal spinal cord N-glycoprofile, we investigated whether an aligned collagen hydrogel could influence the postinjury glycosylation status, using hydrophilic interaction liquid chromatography-ultraperformance liquid chromatography (HILIC-UPLC).

To understand which glycosylation pathways could be important for repair, we chose to investigate glycosylation in a naturally regenerative scenario. For this, we employed an amphibian model, *Xenopus laevis*, as it presents two distinct responses to SCI depending on whether the animal is pre- or postmetamorphic, *i.e.*, regenerative success (tadpole) or regenerative failure (froglet), respectively.^45−47^ This allows for the comparison of successful or failed regeneration within the same species. Again, we focus on glycosylation changes following injury comparing these two developmental stages, here using lectin histochemistry to look at a selection of monosaccharides. No biomaterial treatment was included in this part of the study.

The primary aim of this work was to improve our understanding of the pathology of SCI from a new molecular perspective, and how the injured mammalian spinal cord responds to a biomaterial treatment. We also investigated the mechanism of how the injured spinal cord may be regenerated spontaneously in an amphibian model. In both models, we focused on the glycosylation changes which occur following transection injury, and we would like to bring this knowledge together to improve the design of future biomaterial therapies so that they can be functionalized in a more informed manner.

## Experimental Section

**Figure 1:**
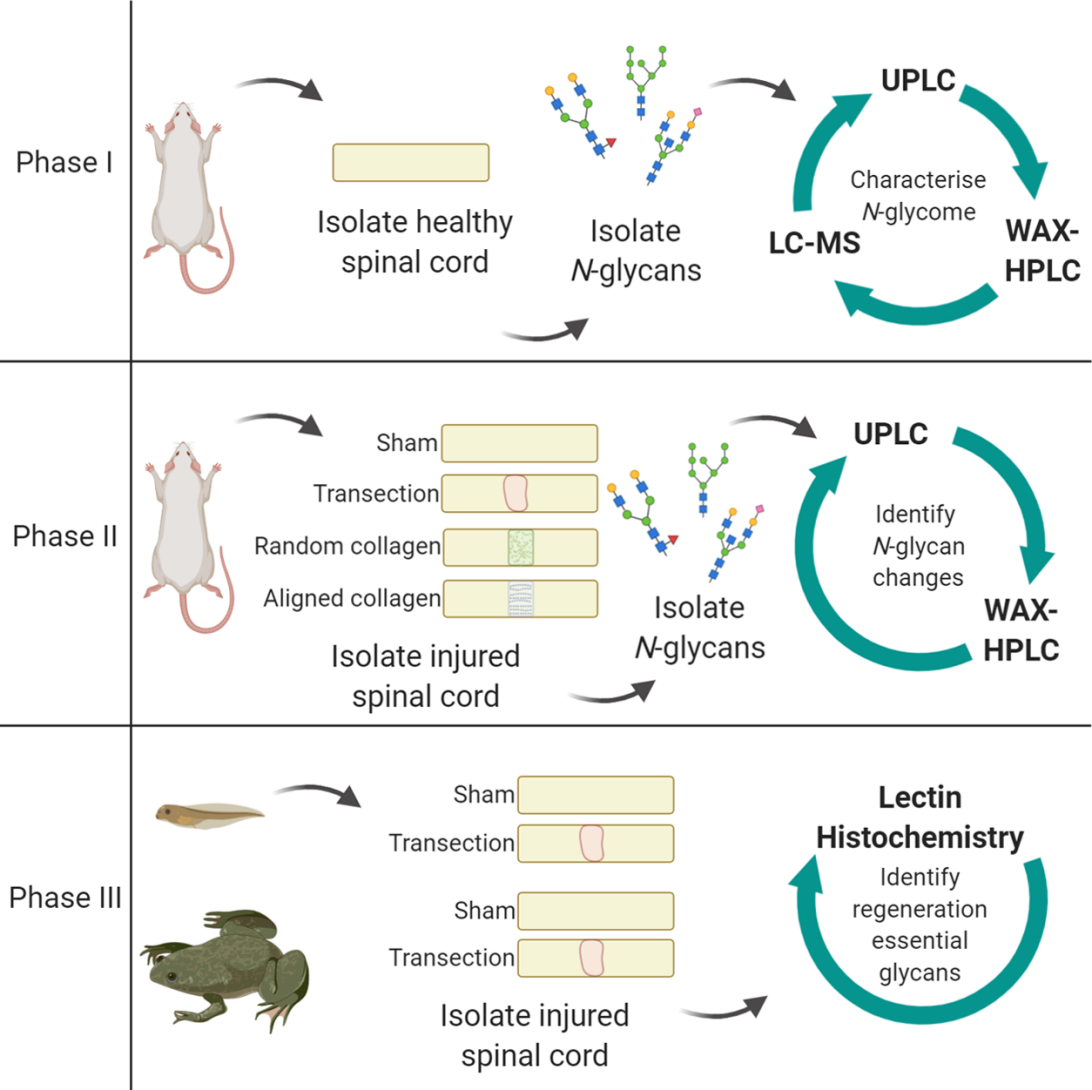
Experimental design. In the first phase, the N-glycome of the adult female rat spinal cord was characterized. Phase II examined how this profile changes in response to spinal cord transection, and treatment with collagen hydrogel in either a random or aligned orientation, at 7 and 14 days post-injury (dpi). The third phase employed *X. laevis* to model injury and compared the glycosylation response in the regenerative premetamorphic stage and the nonregenerative postmetamorphic stage, at 1 and 7 dpi. UPLC, ultrahigh-performance liquid chromatography; WAX-HPLC, weak anion exchange high-performance liquid chromatography; LC-MS, liquid chromatography coupled mass spectrometry. Created with BioRender.com.

The study design is outlined in Figure 1. Briefly, the first phase involved characterization of the N-glycome of the healthy Sprague Dawley female adult rat spinal cord, followed by an investigation into how the N-glycome changes following transection injury in the second phase, and whether this can be modified by treatment with collagen hydrogel with either aligned or randomly oriented fibers. In the third phase, transection injury was performed in pre- and postmetamorphic phases of the frog *X. laevis* to compare the glycosylation response to injury in regeneration permissive or nonpermissive environments.

All *in vivo* experiments involving rat or *X. laevis* were carried out in accordance with the Council Directive 2010/63EU of the European Parliament. All housing and surgical procedures carried out in this study were approved by the Animal Care Research Ethics Committee at the National University of Ireland, Galway, and the Health Products Regulatory Authority.

### SCI Modeling in Rat and *X. laevis*

SCI was modeled in adult female rats with complete transection of the spinal cord. Collagen hydrogels were implanted immediately following transection for the aligned and random collagen hydrogel-treated groups. For sham controls, a laminectomy was performed to expose the spinal cord. Details of surgical procedures, postoperative care, tissue harvesting, and hydrogel preparation can be found in the Supporting Methods.

SCI was modeled in *X. laevis* by complete transection of the spinal cord in both premetamorphic tadpoles (Nieuwkoop and Faber (NF) stage 50) and postmetamorphic froglets (NF stage 66). Sham injured controls had the spinal cord exposed but not transected. No hydrogel treatment was employed in this model. Details of surgical procedures, postoperative care, and tissue harvesting can be found in the Supporting Methods.

### N-Glycan Analysis of the Rat Spinal Cord

Characterization of the N-glycome of healthy spinal cord was performed using a pool of thoracic spinal cord from 14 healthy adult female rats. For all SCI experiments, N-glycosylation experiments were performed using three rats per group, per time point. N-Glycans were enzymatically released from spinal cord homogenates using PNGaseF.^48,49^ Released N-glycans were labeled with 2-aminobenzamide (2AB). HILIC-UPLC was the primary method used to profile the spinal cord N-glycome. A panel of exoglycosidases were used to sequentially remove the terminal monosaccharide residues, and the resulting changes to the profile were compared at each level of digestion to determine the N-glycan composition of the spinal cord. Assignments were supported by liquid chromatography coupled mass spectrometry (LC-MS). Sialylated N-glycans were separated from neutral glycans by WAX-HPLC. The proportion of Neu5Ac and Neu5Gc subtypes of sialic acid was determined with the LudgerTag DMB (1,2-diamino-4,5-methylenedioxybenzene·2HCl) sialic acid release and labeling kit (Ludger).

### Sample Preparation

Frozen rat spinal cord tissue was allowed to defrost on ice. Tissue was placed in a microtube with 2% SDS in 100 mM Tris, pH 6.6, and homogenized in the Qiagen Tissue Lyser II. The homogenate was centrifuged at 13,400 rpm for 20 min at 4 °C. The supernatant was collected and stored at −80 °C.

N-Glycans were released from spinal cord homogenates. Briefly, homogenates were dried in a vacuum centrifuge and incorporated into acrylamide gels. Gels were reduced, alkylated, and washed with acetonitrile and 20 mM NaHCO_3_, pH 7.0, before releasing N-linked glycans overnight at 37 °C with PNGaseF (New England Biolabs) diluted 1:400 in 20 mM NaHCO_3_. Released N-glycans were collected with ultrapure water and acetonitrile and dried completely, before labeling with 2-aminobenzamide (2AB). Glycans were treated with 1% formic acid (Sigma Aldrich, Ireland) and incubated in 2AB labeling solution (2AB, acetic acid, sodium cyanoborohydride, and dimethyl sulfoxide) for 2 h at 65 °C. The excess label was removed by applying samples to Whatman 3MM chromatography paper and washing with acetonitrile. Clean labeled glycans were eluted in water.

### Separation and Detection of N-Glycan Species

All liquid chromatography separations were performed using Waters Acquity UPLC separation modules, Waters temperature control modules, and Waters Acquity fluorescence detectors with an excitation wavelength of 350 nm and emission wavelength of 397 nm.

#### Hydrophilic Interaction Liquid Chromatography-Ultraperformance Liquid Chromatography (HILIC-UPLC) Separation and Quantification of Released, Labeled N-glycans

HILIC-UPLC was used to profile the spinal cord N-glycome, employing an Acquity UPLC glycan BEH amide column, 1.7 μm particle size, 2.1 mm × 150 mm (Waters). Mobile phase (MP) A was 50 mM ammonium formate pH 4.4, and MP B was 100% acetonitrile. A dextran calibration ladder (Waters) was included as an internal standard. The gradient flow rate and temperature are detailed in Supporting Table S1.

### Weak Anion Exchange High-Performance Liquid Chromatography (WAX-HPLC)

For WAX-HPLC, the starting gradient was 100% A, changing to 100% B at 40 min and returning to 100% A at 43 min. The total run time was 50 min per sample, and the flow rate was 0.75 mL/min. The column was kept at room temperature. Samples were resuspended in 50 μL of ultrapure water, and 49 μL was injected.

### DMB Assay

Chemical analysis of the sialic acid subtype was performed using the LudgerTagTM DMB (1,2-diamino-4,5-methylenedioxybenzene·2HCl) sialic acid release and labeling kit (Ludger, U.K.). Spinal cord homogenate was incubated for 1 h in 0.1 M HCl at 80 °C to release sialic acids. The labeling reagent was prepared according to kit instructions, added to hydrolyzed sample and standards, and incubated for 3 h at 50 °C in darkness. The reaction was terminated by the addition of water. Sialic acid subtypes were analyzed by HILIC-UPLC. Flow and gradient details are given in Supporting Table S2. Identity analysis of the sialic acid subtypes was performed by comparing retention times of peaks in the spinal cord sample to the retention times of the reference standards.

### Liquid Chromatography Coupled Mass Spectrometry (LC-MS)

LC-MS was performed using a Waters Xevo G2 QToF spectrometer coupled to a Waters Acquity UPLC system. The same column and MPs were used for HILIC-UPLC and LC-MS.

N-Glycans were released and labeled as above. Excess 2AB was removed by solid-phase extraction with normal phase amide resin PhyTips (PhyNexus, California). Excess 2AB was removed with acetonitrile, and glycans were eluted in 20% acetonitrile and dried completely. LC-MS was performed using a Waters Xevo G2 QToF spectrometer coupled to a Waters Acquity UPLC system. The gradient is shown in Supporting Table S3. Spinal cord glycan samples were resuspended in 75% acetonitrile for LC-MS analysis. MassLynx software was used for both instrument control and subsequent analysis. The sample infusion rate into the mass spectrometer was 5 μL/min, and the samples were ionized by electrospray ionization. The experiment was run in negative and sensitivity modes for optimal detection of 2AB-labeled glycans. Leucine enkephalin (Waters) was used as a reference compound for lock mass correction. Parameters for the mass spectrometer are given in Supporting Table S4.

### Exoglycosidase Digestions

N-Glycan samples were digested with a panel of exoglycosidase enzymes in 50 mM sodium acetate overnight at 37 °C (Table 1). Enzymes were inactivated by heating to 65 °C for 15 min. Digested glycans were separated from enzymes with 10 kDa molecular weight cut-off micro-centrifuge filtration devices (Pall Life Sciences) and dried completely *via* vacuum centrifugation.

**Table 1:** Details of Exoglycosidase Enzymes and Digestions^a^

enzyme	full name	specificity	supplier	cat no.	units (U/mL)	volume (μL)
ABS	arthrobacter ureafaciens sialidase	α(2-3, -6, -8) linked sialic acid	NEB	P0722	20,000	1
NAN-1	recombinant sialidase	α(2-3) linked sialic acid	NEB	P0743	50,000	1
BTG	bovine testes β-galactosidase	β(1-3, -4) linked galactose	Prozyme	GKX-5013	5	2
SPG	Streptococcus pneumoniae β-galactosidase	β(1–4) linked galactose	Prozyme	GKX-5014	2	2
CBG	coffee bean α-galactosidase	α(1–3, -4) linked galactose	Prozyme	GKX-5007	5	2.5
AMF	almond meal α-fucosidase	α(1–3) linked fucose (outer-arm fucose)	NEB	P0769	4,000	1
BKF	Bovine kidney α-fucosidase	α(1–6) linked fucose (core fucose), some α(1-2) outer-arm fucose	NEB	P0749	2,000	1
GUH	*Streptococcus pneumonia* hexosamindase	β-linked GlcNAc	Prozyme	GK800050	40	1
JBH	Jack bean β-*N*-acetylhexoaminidase	β-linked GlcNAc and GalNAc residues	Prozyme	GKX-5003	50	2
JBM	Jack bean mannosidase	α(1-2, -6, -3) linked mannose	NEB	P0768	2,000	2
enzyme combinations						
1	UND, Undigested, no enzyme					
2	NAN-1					
3	ABS					
4	ABS + BTG					
5	ABS + BTG + BKF					
6	ABS + BTG + AMF					
7	ABS + AMF					
8	ABS + BTG + BKF + AMF					
9	ABS + BTG + BKF + AMF + GUH					
10	ABS + BTG + BKF + AMF + GUH + CBG					
11	ABS + BTG + BKF + AMF + GUH + JBM					
12	ABS + SPG					
13	ABS + JBH					

a Digestions were performed in a total of 10 μL and in the presence of 0.05 M sodium acetate buffer (1 μL). Digestion with JBM was performed independently, in the presence of Zn^2+^ (0.1%), following ABS + BTG + BKF + GUH digestion on the previous day. NEB, New England Biolabs.

### Lectin- and Immunohistochemistry

Glycosylation changes in *Xenopus* were investigated using lectin histochemistry. A dual staining protocol was employed where FITC-conjugated lectins were incubated on tissue sections, followed by incubation with primary and secondary antibodies. Lectins were purchased from EY labs and included SNA-I, which binds terminal α(2–6) sialic acid (10 μg/mL), DSA, which binds terminal GlcNAc (5 μg/mL), and WFA, which binds terminal GalNAc (10 μg/mL). Primary antibodies included (mouse anti-CD11b (EMD Millipore, 1:200), rabbit anti-GFAP (Dako, 1:400), and mouse anti-β-III Tubulin (Millipore, 1:200)) Sections were counterstained with Hoechst (Invitrogen, 1:2000). A similar labeling procedure was used for rat tissue sections. All images were acquired with an Andor Revolution spinning disk confocal microscope (Andor Technology Ltd) at 40x magnification (see Supporting Figure S1) and analyzed in Fiji (version 1.50d, National Institute of Health. Java 1.60_24 (64-bit)), see Supporting Methods.

### Statistical Analysis

N-Glycan results were analyzed in Minitab 17 using the general linear model analysis of variance, and Tukey’s multiple comparisons test with *p* < 0.05 considered statistically significant.

Lectin histochemistry experiments in *X. laevis* were analyzed using GraphPad Prism software version 5.0 (Prism 5). Differences between sham and transection groups were investigated with the Student *t*-test. Comparisons across time point and developmental stage were investigated using two-way ANOVA followed by Bonferroni’s post hoc test, with *p* < 0.05 considered statistically significant.

All results were presented as mean ± standard error of the mean (SEM).

## Results

### N-Glycan Composition of the Healthy Adult Female Rat Spinal Cord

HILIC-UPLC with exoglycosidase digestion was used to identify the N-glycan species present in the rat spinal cord. A HILIC-UPLC profile was obtained for each combination of exoglycosidase enzymes and compared back to the undigested (UND) profile to identify the glycans present.

**Figure 2:**
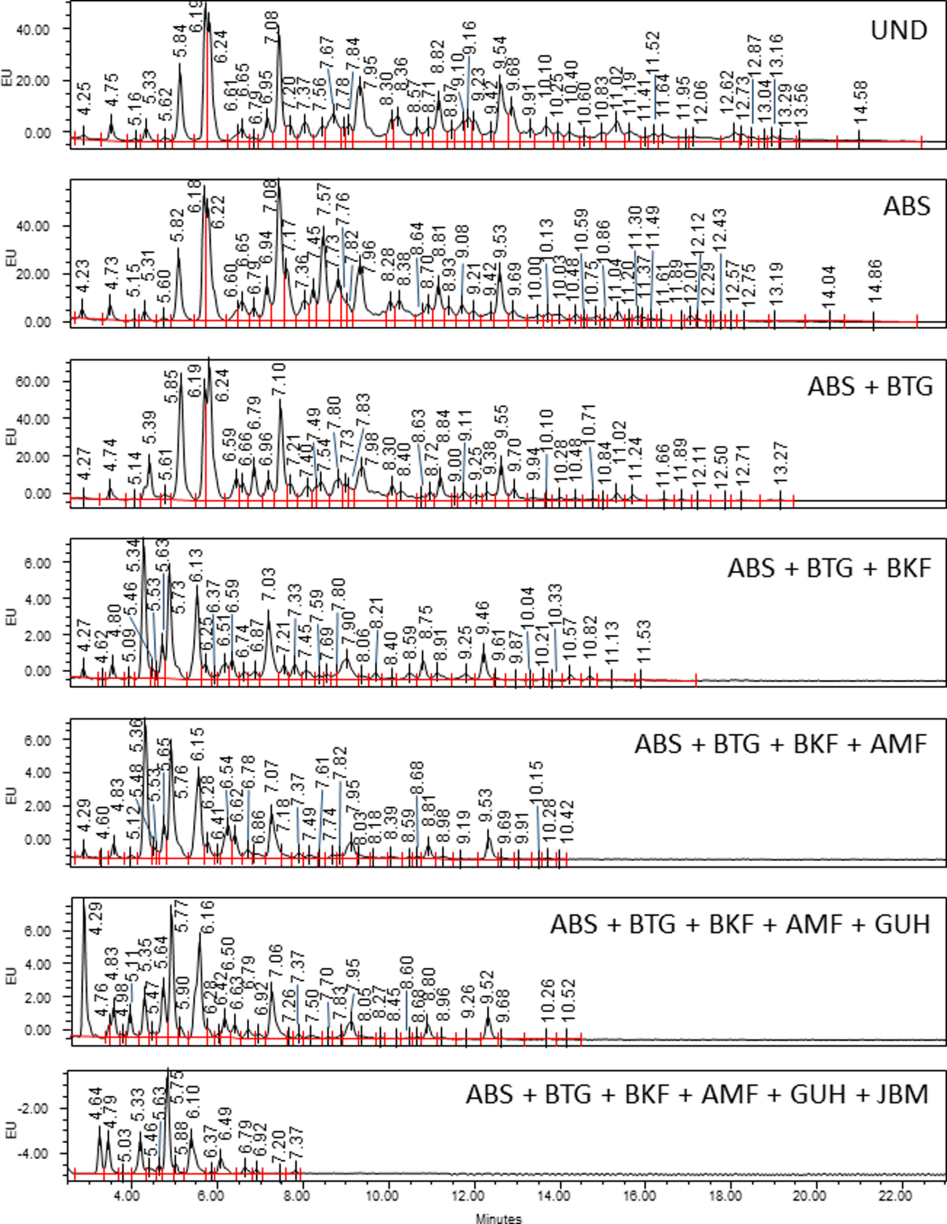
Main panel of exoglycosidase digestions profiled using HILIC-UPLC. ABS removes α(2–3, 6, 8)-sialic acids, BTG removes β(1–3, 4) galactose, BKF removes core α(1–6)-fucose and outer-arm α(1–2)-fucose, AMF removes α(1–3, 4)-fucose, GUH removes β-GlcNAc, and JBM removes mannose. Peaks are labeled with GU values. EU, emission units.

Clear shifts can be seen with ABS (sialidase), BTG (galactosidase), BKF and AMF (fucosidases), and GUH (hexosaminidase), indicating that sialic acids, galactose, and fucose decorate multiantennary glycans (Figure 2).

Digestion of the glycans with JBM (mannosidase) indicates a large abundance of mannose species also. Supporting Table S5 indicates the primary structures for each peak. Linkage analysis chromatograms are shown in Supporting Figure S2. The full complement of all structures identified in the undigested sample and for each enzyme digest is shown in Supporting Table S6, and mass data for those structures confirmed by LC-MS are in Supporting Table S7.

**Figure 3:**
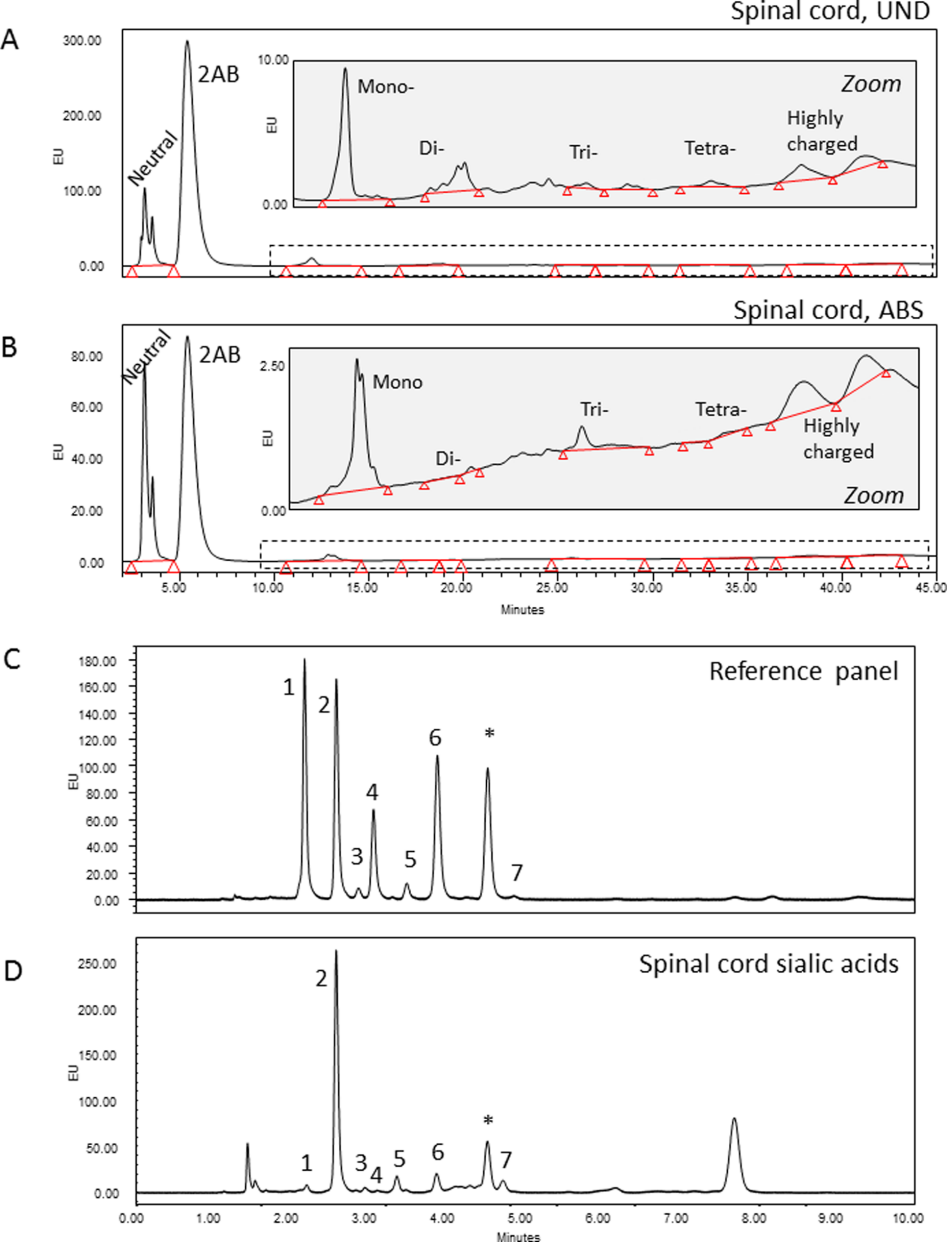
Sialic acids of rat spinal cord. WAX-HPLC separates glycans on the basis of charge, and fetuin-*N* was used as a reference standard. Neutral N-glycans (77%); mono-charged (12%); di-charged (4%); tri-charged (1%); tetra-charged (1%); unidentified highly charged N-glycan species (5%); 2AB, excess 2AB label; (A) UND, undigested; (B) ABS, ABS (sialidase) digested. The dashed line indicates the zoomed region. (C) Reference standard for DMB analysis. (D) Neu5Ac is the most common type of sialic acid in the rat spinal cord, with minor amounts of Neu5Gc- and Neu5xAc2-type sialic acids. Sialic acids were released from spinal cord glycans by hydrolysis and separated on the basis of chemical structure. Peak 1, Neu5Gc; peak 2, Neu5Ac; peak 3, Neu5,7Ac2; peak 4, Neu5Gc9Ac; peak 5, Neu5,8Ac2; peak 6, Neu5,9Ac2; and peak 7, Neu5,x,xAc3, where x is an unknown position; * contaminant peak. Note that DMB analysis was performed on total spinal cord glycans, and WAX-HPLC on N-glycans.

Weak anion exchange (WAX)-HPLC was used to separate the N-glycans of the spinal cord based on charge (Figure 3A,B). In general, sialic acid is the leading donor of charge to any N-glycan. The majority (77%) of N-glycans present were neutral, *i.e.*, without sialic acid. There were small proportions of mono-sialylated (12%) and di-sialylated (4%) species present also, with a minimal amount of tri- (1%) and tetra-sialylated glycans identified (<1%) (Figure 3C). Sialidase digestion increased the area of the neutral peak (S0) to 86%, indicating that 10% of charged species were due to unmodified sialic acid (Figure 3B). Other charged species might be present, such as sulfate or phosphate groups, chains of polysialic acid, or other acidic carbohydrates such as glucuronic acid. The presence of such groups inhibits exoglycosidase digestion. Sulfate groups have been identified in spinal cord N-glycans, as well as sialic acid carrying extra acetyl groups (Supporting Tables S6 and S7). Phosphate groups or glucuronic acid were not detected. The highly charged region at the end of the profile was unaffected by ABS digestion and so did not contain unmodified sialic acids.

DMB assay demonstrated that the majority of spinal cord sialic acids were present as Neu5Ac type, with minor amounts of Neu5Gc and Neu5Ac with one or two extra acetyl groups (Supporting Figure S3D). It should be noted that the DMB assay is performed on total spinal cord sialic acids, not just those present on N-glycans; however, LC-MS analysis identified sialic acids of Neu5Gc, Neu5Ac and Neu5xAc2, and Neu5xxAc3 types on spinal cord N-glycans (Supporting Table S7).

**Figure 4:**
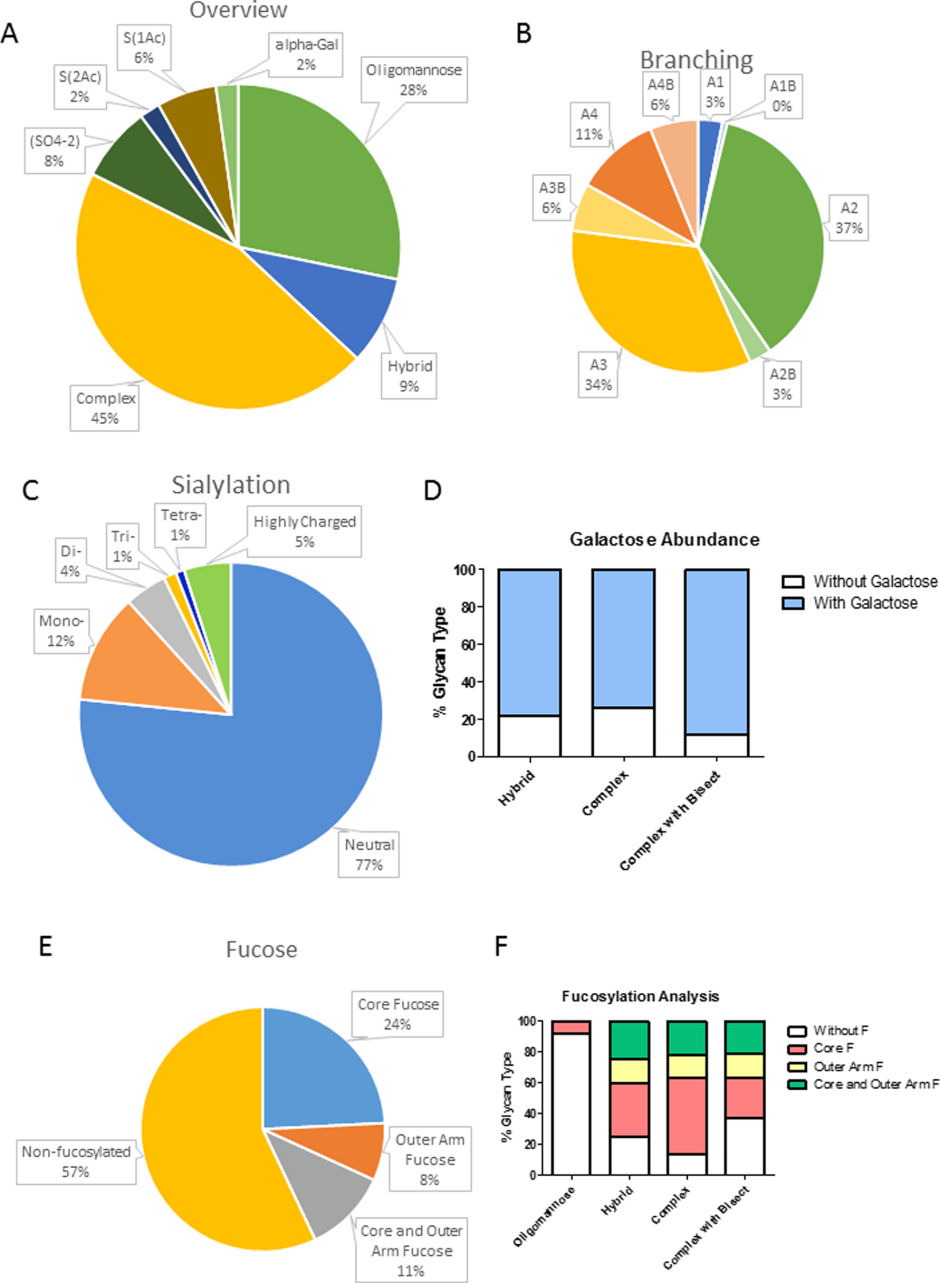
Summary of the types of N-glycan identified in the healthy adult rat spinal cord. (A) All of the major classes of N-glycan can be found in the spinal cord, oligomannose, hybrid, and branched. Branched glycans also occur with a bisecting GlcNAc β(1–4) linked to the central mannose. Some more unusual features such as acetylated sialic acids, sulfate groups, and α-linked galactose can also be found on complex or hybrid glycans. (B) Number of branches on complex glycans, some also feature a bisecting GlcNAc β(1–4) linked to the central mannose. A2 and A3 structures are most common. (C) Charge abundance as calculated using WAX-HPLC. (D) Extent of branch elongation with galactose on each class of N-glycan. (E) Proportion of total glycans decorated with both core and outer-arm fucose. (F) Distribution of fucosylation across the main N-glycan classes.

Feature analysis involves grouping individual N-glycan species according to their main monosaccharide constituents and gives us a broader understanding of the types of glycans present. The majority of N-glycans identified were found to be complex (45%), being mono-, di-, tri-, or tetra-antennary. Some of these carried a bisecting GlcNAc. Some complex glycans carried unusual glycan features including sulfate groups (8%), sialic acids modified with extra acetyl groups (8%), and α-galactose containing glycans (2%) (Figure 4A). Of the complex glycans, di- and tri-antennary were most common (A2 and A3) contributing 37 and 34% of total N-glycans (Figure 4B). On complex glycans extension of the branch with galactose residues was widespread (Figure 4D). Galactose was found to be present mainly in β(1–4) linkage to the underlying GlcNAc (Supporting Figure S2). A range of high-mannose species were also highly abundant, having from 3 to 9 mannose residues and making up approximately 28% of all N-glycans (Figure 4A). Large high-mannose structures, M6, M7, M8, M9, and M9Glc1, were far more abundant than the smaller M4 and M5 species. M4 and M5 were more commonly found as hybrid glycans, *i.e.*, oligomannose structures with a single GlcNAc antenna. These constituted 9% of all N-glycans (Figure 4A).

Fucosylation was found to be very common on rat spinal cord N-glycans. Core fucose was present on 24% of all N-glycans, outer-arm fucose was seen on 8% of all N-glycans, and 11% of all N-glycans were decorated with both core and outer-arm fucose residues (Figure 4E). Fucose was similarly distributed across the hybrid, complex, and bisected glycans (Figure 4F). Of the oligomannose species, the majority were nonfucosylated, with only 8% containing core fucose (Figure 4E). Outer-arm fucose is not attached to oligomannose glycans.

### Spinal Cord Transection Disrupts the N-Glycosylation Profile of the Rat Spinal Cord

**Figure 5:**
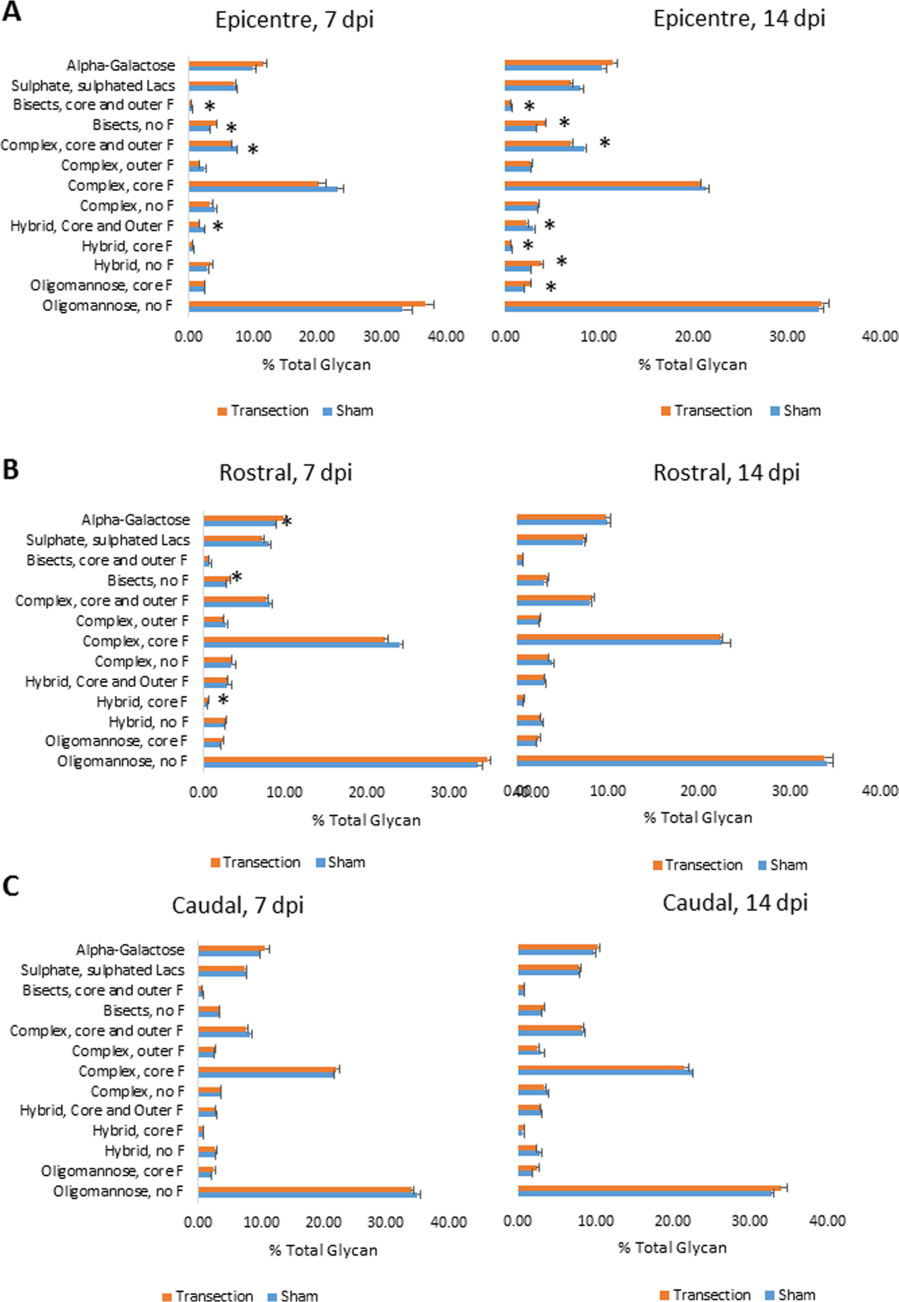
Differences in N-glycan features between sham and transection-only groups, at 7 and 14 dpi in the rat spinal cord. (A) Lesion epicenter, (B) rostral to injury, and (C) caudal to injury. Asterisks indicate a statistically significant difference between sham and transected groups, using the *t*-test, *n* = 3, *p* < 0.05.

The major glycan for each peak (indicated in Supporting Table S5) was considered when examining changes in glycosylation following SCI. Transection of the spinal cord resulted in alteration in the abundance of particular N-glycan features at the lesion epicenter. This could be seen as early as 7 dpi and was maintained mainly at 14 dpi (Figure 5A). Comparing transected to the sham injured spinal cord, there were some small but significant changes (Figure 5). In the lesion epicenter at 7 dpi, there was a decrease in all complex-type N-glycans except α-galactosylated and nonfucosylated bisects and statistical significance was reached in four of these nine groups (Figure 5A). There was a corresponding increase in oligomannose N-glycans. By 14 dpi, there was a similar reduction in complex N-glycans, but oligomannose structures were approximately equal between sham and transection groups with the increase seen in hybrid and core-fucosylated oligomannose glycans instead (Figure 5A). There was a loss of fucose on bisected glycans: fucosylated bisected glycans were significantly reduced, and those carrying no fucose were significantly increased at both 7 and 14 dpi (Figure 5A). In the transection-only group, there were some changes over time: complex glycans carrying outer fucose and hybrid glycans carrying both core and outer fucose increased significantly between 7 and 14 dpi in this group (Figure 5A).

This alteration of the N-glycosylation profile was primarily a local response, with a few changes seen on either side of the injury epicenter (Figure 5C). However, rostral to the injury (5–15 mm from the lesion epicenter) at 7 dpi, there were significant increases in glycans featuring α-linked galactose bisects without any fucose and hybrids with core fucose following SCI (Figure 5B). By 14 dpi, these differences had resolved.

### Minor Modification of the Glycosylation Injury Response Occurs Following Implantation of Collagen Hydrogel

**Figure 6:**
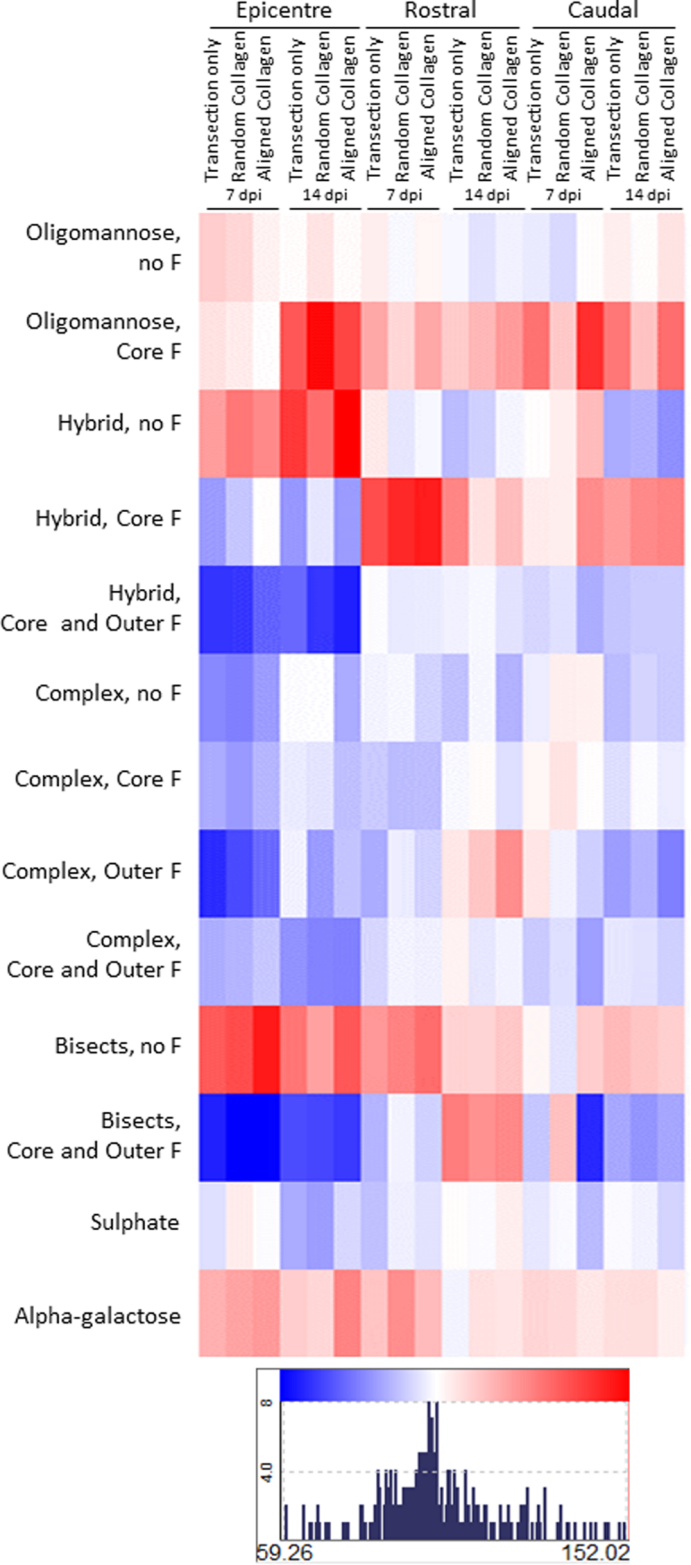
Changes in N-glycans in the lesion epicenter, rostral and caudal to injury of rat spinal cord with collagen hydrogel treatment. Individual GPs were grouped according to whether they were high-mannose, hybrid, complex, or complex with bisect, and the type of fucose (F) they were decorated with. Data presented here are expressed as the percentage of the sham group for the appropriate time point, *n* = 3. Variance within groups was low, with RSD being ≤10% in most cases and ≤20% in the remainder. Blue squares indicate a reduction compared to sham, with the greatest reduction shown in dark blue at 59.26%. Red squares indicate an increase compared to sham, with the greatest increase shown in dark red at 152.02%.

Collagen hydrogel treatment following transection injury had little impact on the spinal cord N-glycoprofile. Normalizing data to the sham group, there were very few differences between transection-only and either collagen hydrogel-treated group in any region of interest (ROI) and none were statistically significant (Figure 6). From the heat map shown in Figure 6, it can be seen that very similar amounts of each glycan type were present in the injured cord regardless of treatment. The transection injury itself was the primary contributor to altered glycosylation profiles. In all three ROIs, the ANOVA test found time point to be the significant source of variability for many glycan types, suggesting that the glycosylation response is a dynamic one. Specifically, in the lesion epicenter, the amount of core-fucosylated oligomannose structures increased significantly between 7 and 14 dpi in those animals treated with random collagen hydrogel post-SCI, while sulfated glycans were reduced in this group over the same time period (Figure 6). Aligned collagen hydrogel had an influence on complex glycans carrying both core and outer fucose, which were significantly reduced at 14 dpi compared to the earlier time point (Figure 6). For many glycan types (including oligomannose with core fucose, a-fucosylated complex glycans, branched glycans with core and/or outer fucose, and bisected glycans with and without fucose) when the three injured groups were considered together, there were significant differences between the 7 and 14 dpi time points (Figure 6).

### Charged Species Are Increased following SCI, and Sialic Acid Is Associated with Inflammatory Cells

Since sialylated structures are relatively minor components of the N-glycome of the intact spinal cord, changes in these potentially essential species may be masked by more abundant structures. WAX-HPLC was performed on N-glycan samples from the lesion epicenter of each experimental group to provide a more in-depth analysis of changes in charged structures. Supporting Figures S3 and S4A,B show representative undigested WAX-HPLC profiles for each experimental group at 7 and 14 dpi. At 7 dpi in all injured groups, there was an increase in charged species in the spinal cord (Supporting Figure S4C). Di-sialylated and tri-sialylated (S2 and S3) were increased in comparison to sham. At 14 dpi, the aligned collagen hydrogel group also seemed to have increased charged structures, in particular S1, S2, and S3 (Supporting Figure S4C). No statistics were performed on these data due to low n numbers.

**Figure 7:**
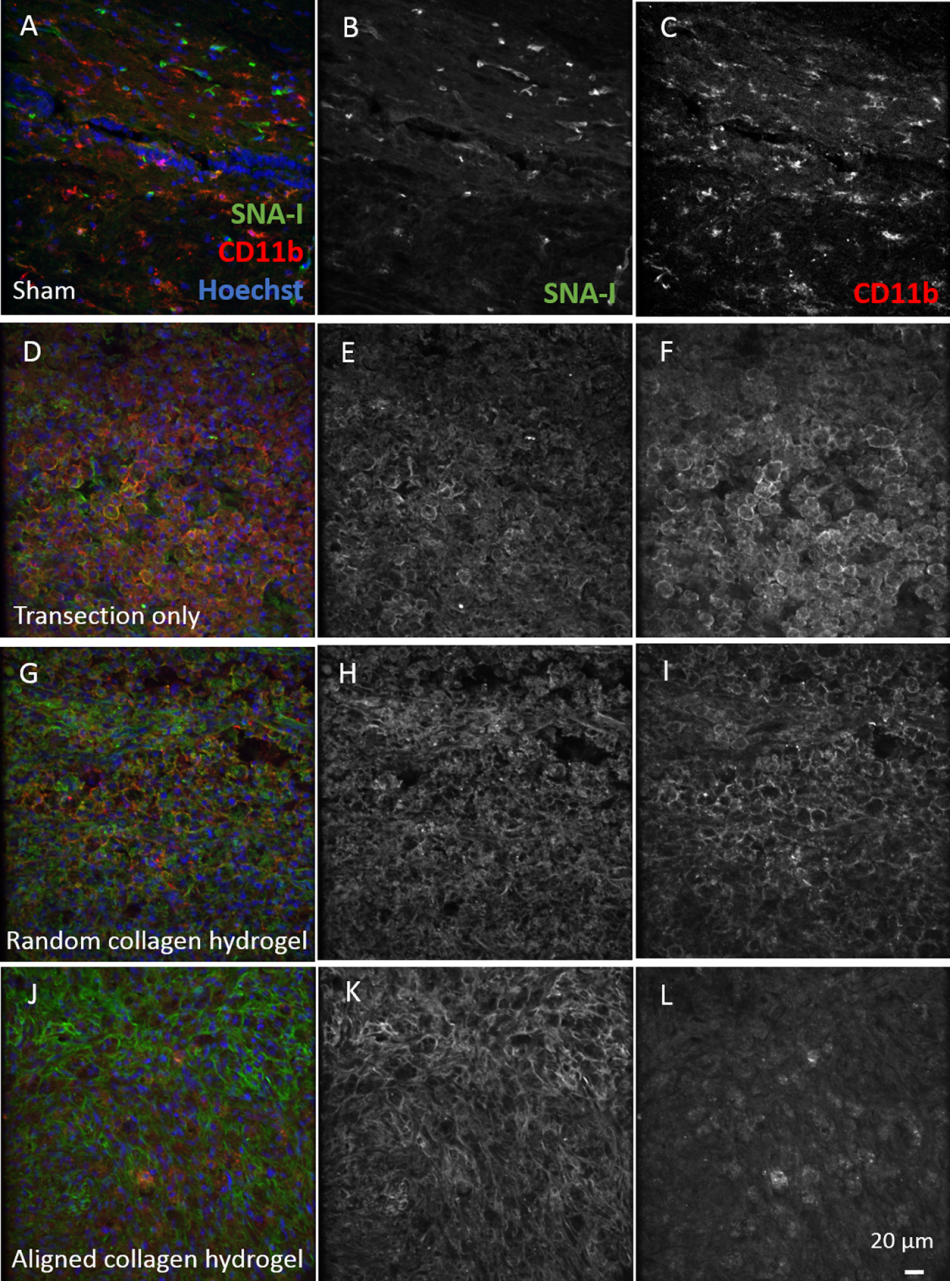
Distribution of sialic acid labeled with SNA-I lectin and its relationship to CD11b-positive microglia and macrophages in the rat spinal cord. (A–C) Sham (D–F) transection-only, (G–I) random collagen hydrogel-treated, and (J–L) aligned collagen hydrogel-treated. SNA-I single-channel images are shown for each group in (B, E, H, K); CD11b single-channel images are in (C, F, I, L). Images from injured animals (D–L) were captured at the borders of the injury. All images are from the spinal cord at 7 dpi. Scale bar is 20 μm.

Since sialic acid has been implicated in the regulation of inflammation, lectin histochemistry with the SNA-I lectin, which binds α(2–6)-sialic acid, was performed in combination with immunohistochemistry against CD11b, a marker of macrophages and microglia, at 7 dpi (Figure 7). In sham tissue, SNA-I bound what appeared to be blood vessels and the small number of CD11b-positive microglia did not co-localize with positive SNA-I signals at all (Figure 7A). However, in the transected spinal cord, large numbers of CD11b-positive macrophages and microglia were evident at the lesion border, and almost all of the CD11b signal was seen in close proximity to SNA-I lectin signal (Figure 7B). A similar result was seen in the random collagen hydrogel-treated group (Figure 7D). In the aligned hydrogel group, there were still many cells with positive SNA-I binding on their surface. However, the level of positive CD11b staining was lower and did not co-localize with the lectin (Figure 7F). In the lesion epicenter of the transected and collagen-treated groups, there were far fewer CD11b-positive cells, and there was no co-localization between them and the SNA-I lectin (Figure 7C,E,G). SNA-I lectin did not co-localize with astrocytes (Supporting Figure S5A–D) or neurons (Supporting Figure S5E–H) at the borders of the injury or in the sham injured spinal cord. No GFAP-positive or β-III tubulin-positive cells could be detected in the lesion epicenter.

### Regenerative and Nonregenerative Stages of *X. laevis* Differ in Their Glycosylation Response to Spinal Cord Transection

To investigate whether glycosylation patterns differ with regenerative potential following SCI, the response to spinal cord transection was investigated in *X. laevis* using lectin histochemistry. These frogs exhibit regenerative success before metamorphosis and regenerative failure after metamorphosis in response to SCI.^45−47^ A variety of regions were investigated, including the lesion epicenter, the rostral and caudal borders of the lesion, and intact tissue, both rostral and caudally. The lesion epicenters of froglet spinal cords were lost during tissue preparation and were not included. To compare tadpole and froglet responses at 1 and 7 dpi, the data for transected animals were normalized to the relevant sham group so that 100% was the equivalent to the sham response at that time point. Lectins generally bind to the terminal monosaccharide of a glycan. The lectins chosen for this study were picked to examine some key features of N-glycans, which are considered one of the most extensive post-translational modifications on proteins,^50^ and also to give an indication of the level of chondroitin sulfate proteoglycans (CSPGs) present in the tissue, which have been established as essential inhibitors of regeneration in mammalian SCI.^51^

**Figure 8:**
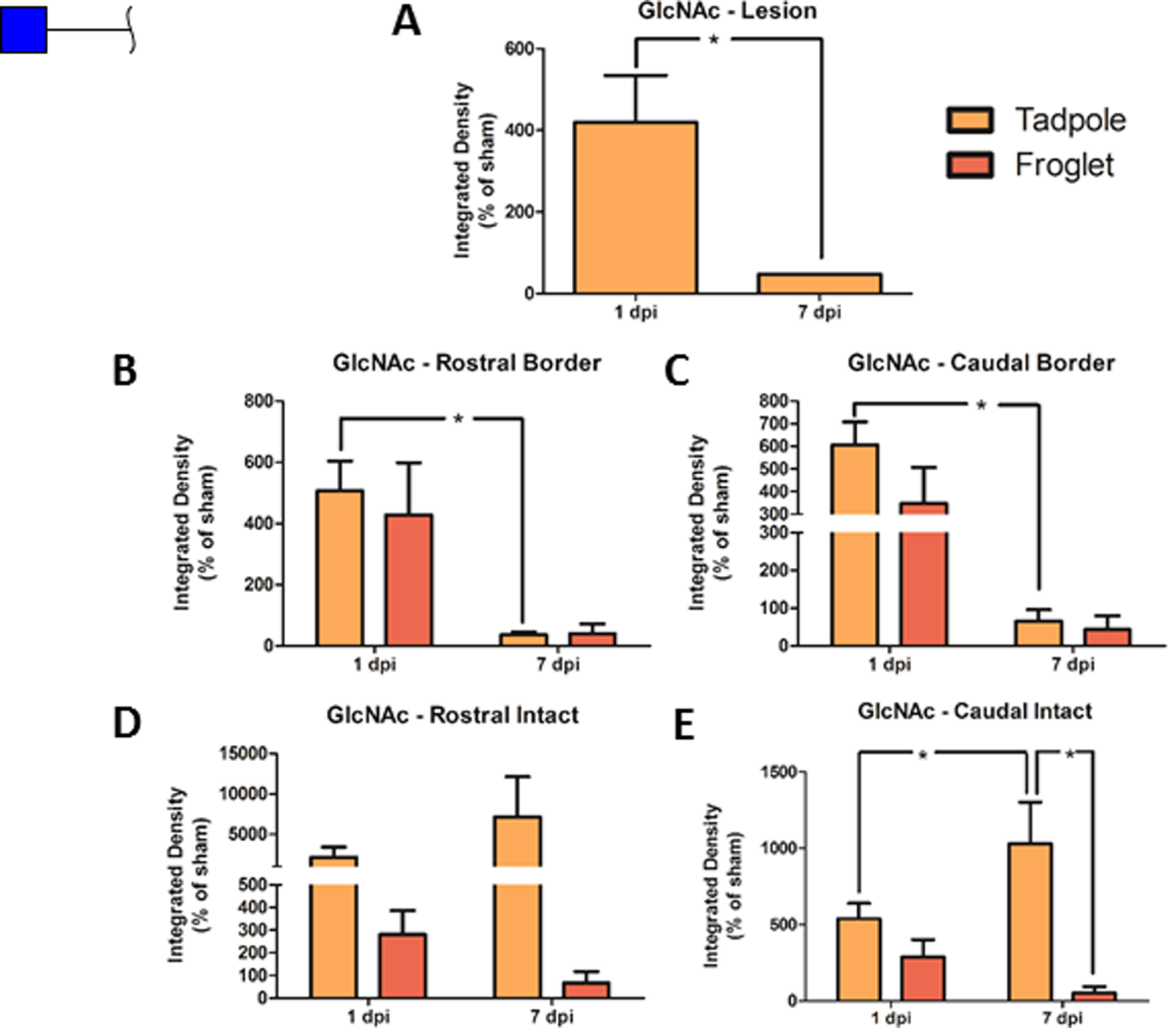
Changes in GlcNAc following SCI in pre-and postmetamorphic *X. laevis*. Graphs show DSA lectin staining in spinal cord tissue in tadpole lesion epicenter (A) and in tadpole and froglet lesion borders rostral (B) and caudal (C) and in intact tissue rostral (D) and caudal (E) at 1 and 7 dpi. Data were normalized to sham before analysis with two-way ANOVA and Bonferroni’s post hoc test, and are presented as mean ± SEM. A value of *p* < 0.05 was considered significant. Groups that differ significantly are indicated with an asterisk.

Terminal GlcNAc was investigated with the DSA lectin. There was a significant increase in GlcNAc following transection of tadpole spinal cord at 1 dpi compared to sham. Comparing developmental stage and time point, a pattern of early increased GlcNAc at 1 dpi followed by a decrease at 7 dpi was observed for both tadpole and froglet, with the statistical difference between the two time points reaching only in the tadpole spinal cord (Figure 8B,C). At the tadpole lesion epicenter, there was also an increase at 1 dpi with a significant reduction by 7 dpi (Figure 6A). In the intact tissue far from the injury (Figure 8D,E), there was significantly more GlcNAc in the tadpole at 7 dpi than at 1 dpi in tadpole and at 7 dpi in froglet at the caudal side. Representative images of DSA staining for all groups and all ROIs are shown in Supporting Figure S7.

**Figure 9:**
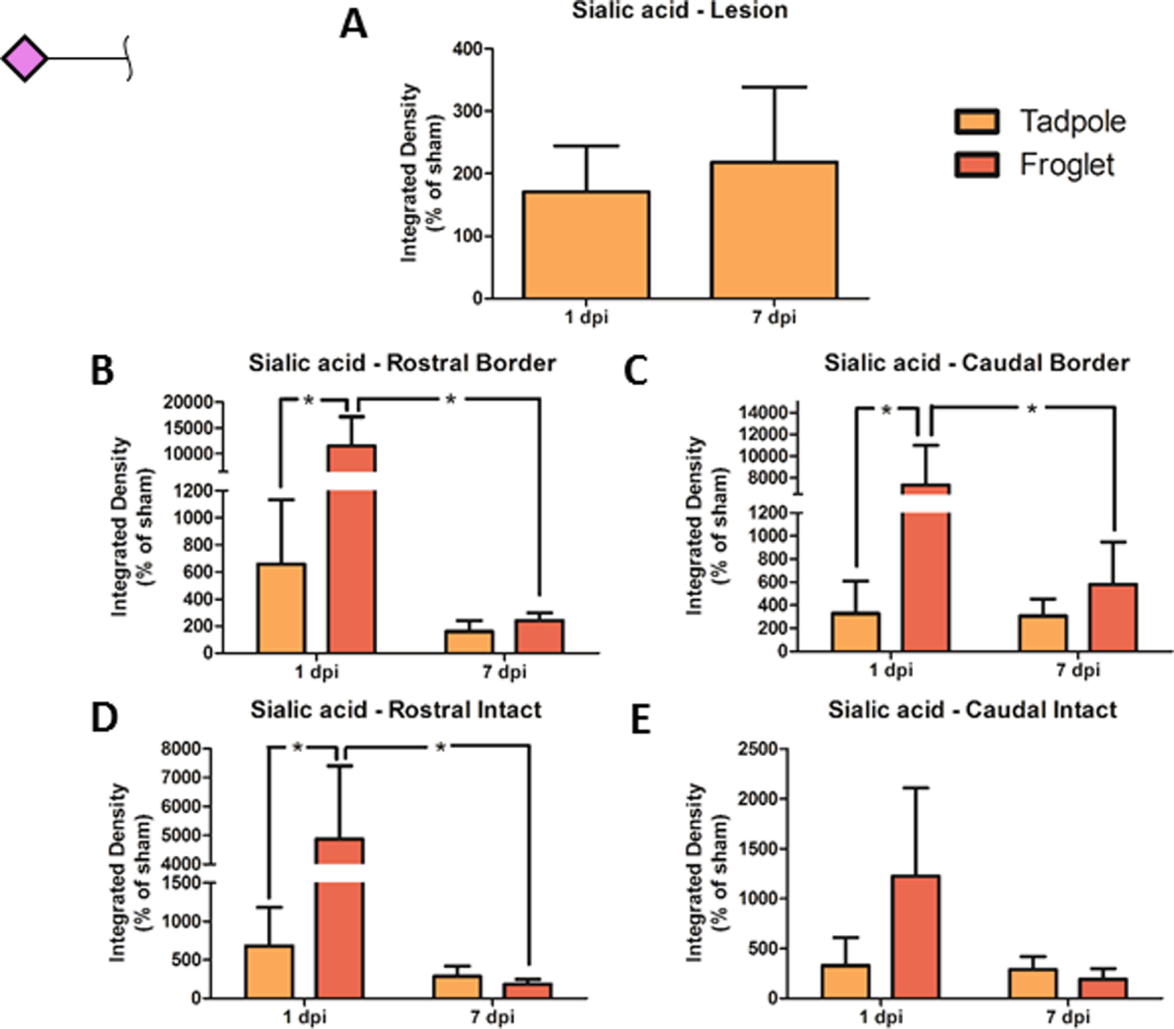
Changes in sialic acid following SCI in pre- and postmetamorphic *X. laevis*. Graphs show SNA-I lectin staining in spinal cord tissue in tadpole lesion epicenter (A) and in tadpole and froglet lesion borders rostral (B) and caudal (C) and in intact tissue rostral (D) and caudal (E) at 1 and 7 dpi. Data were normalized to sham before analysis with two-way ANOVA and Bonferroni’s post hoc test, and are presented as mean ± SEM. A value of *p* < 0.05 was considered significant. Groups that differ significantly are indicated with an asterisk.

The amount of terminal sialic acid (SNA-I lectin staining) present in froglet spinal cord at 1 dpi was significantly higher than in the tadpole spinal cord at 1 dpi at the lesion borders both rostral and caudal and was significantly reduced by 7 dpi (Figure 9B,C). This same pattern was seen in the intact tissue far from the lesion, but the difference was only significant on the rostral side (Figure 9D,E). At the tadpole lesion epicenter, there was no difference in sialylation between 1 and 7 dpi (Figure 9A). Representative images of SNA-I staining for all groups and all ROIs are shown in Supporting Figure S8.

**Figure 10:**
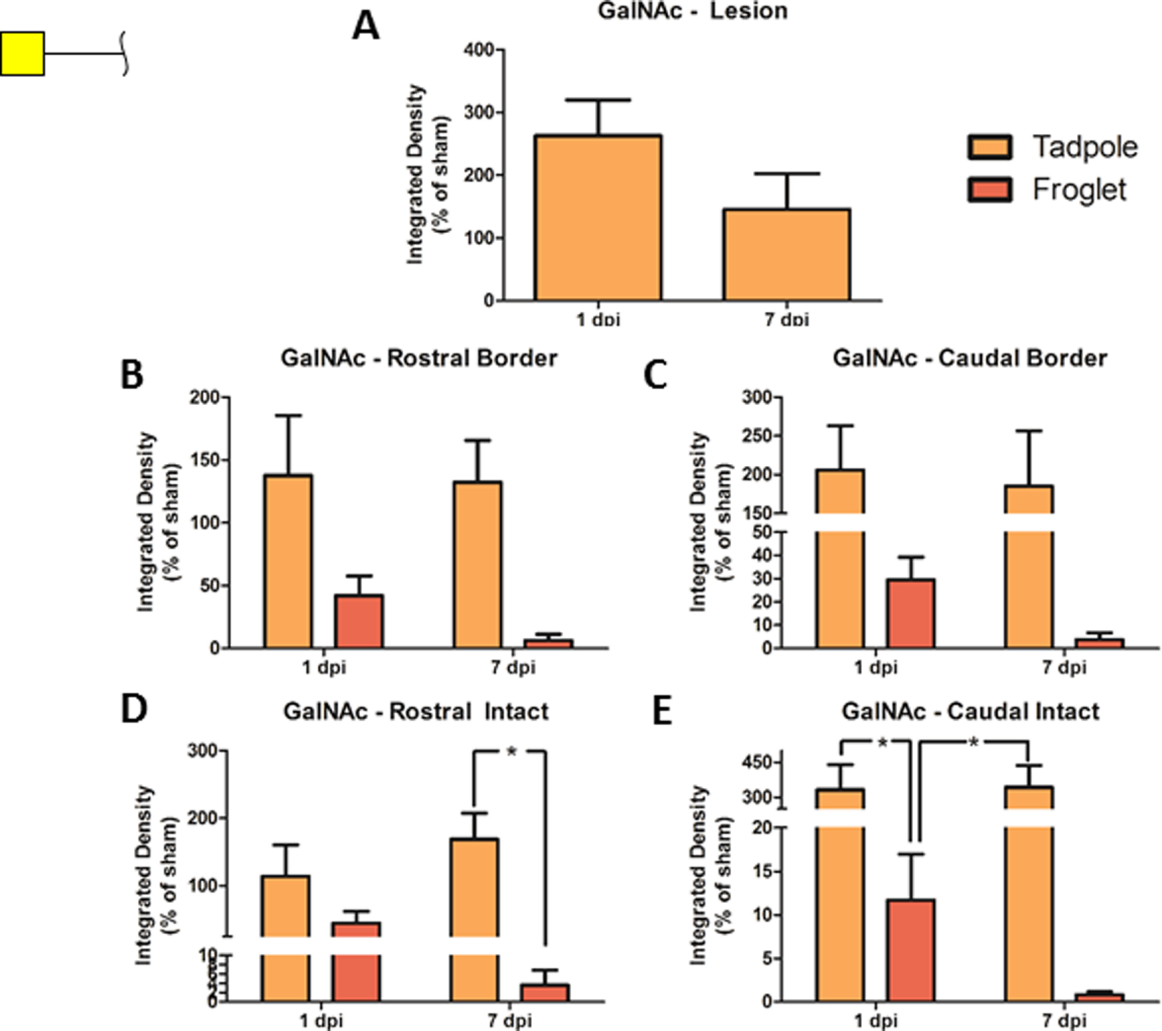
Changes in GalNAc following SCI in pre- and postmetamorphic *X. laevis*. Graphs show WFA lectin staining in spinal cord tissue in tadpole lesion epicenter (A) and in tadpole and froglet lesion borders rostral (B) and caudal (C) and in intact tissue rostral (D) and caudal (E) at 1 and 7 dpi. Data were normalized to sham before analysis with two-way ANOVA and Bonferroni’s post hoc test, and are presented as mean ± SEM. A value of *p* < 0.05 was considered significant. Groups that differ significantly are indicated with an asterisk.

Comparing terminal GalNAc (WFA lectin staining) in tadpole and froglet across each time point, it can be seen that the regenerative tadpoles increased the amount of GalNAc in response to transection and that there was very little change over the first 7 days following the injury (Figure 10). While tadpole spinal cord increased GalNAc production, froglet spinal cord decreased GalNAc at both time points following transection (Figure 10B–E). There appeared to be a large difference between tadpole and froglet, but no statistical significance was seen at the lesion borders (Figure 10B,C). In the intact tissue, however, the amount of GalNAc present was significantly lower in froglet spinal cord than in tadpole for rostral tissue at 7 dpi, and for caudal tissue at both 1 and 7 dpi (Figure 10D,E). GalNAc expression in the lesion epicenter of tadpoles reduced from 1 to 7 dpi but remained above sham levels of expression, and no significant difference was detected (Figure 10A). Representative images of WFA staining for all groups and all ROIs are shown in Supporting Figure S9.

**Figure 11:**
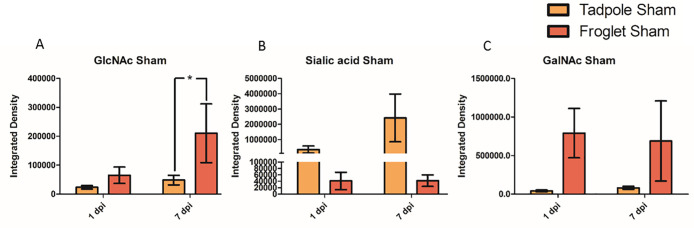
Changes in monosaccharide abundance following sham injury to the spinal cord in pre- and postmetamorphic *X. laevis* at 1 and 7 dpi. Graphs show lectin staining in sham injured tissue at 1 and 7 dpi in tadpole and froglet. (A) GlcNAc, (B) sialic acid, and (C) GalNAc. Data were analyzed by two-way ANOVA and Bonferroni’s post hoc test, and are presented as mean ± SEM. For all tests, a value of *p* < 0.05 was considered significant. Groups that differ significantly are indicated with an asterisk.

To investigate whether the differential response to the injury may be due to baseline differences in the glycan expression at each developmental stage, the sham data were compared for each monosaccharide in tadpole and froglets at 1 and 7 dpi. The developmental stage was found to be a significant contributor to terminal GlcNAc level, where a significant difference was observed in DSA staining between tadpole and froglet sham groups at 7 dpi (Figure 11A). SNA-I staining indicated a difference between tadpole and froglet α(2–6) linked sialic acid, but for this glycan, tadpole tissue produced more sialic acid than froglet, and tadpole sialylation increased over time (Figure 11B). Investigating GalNAc levels in sham tissue with WFA showed that froglet tissue produced more GalNAc than tadpole tissue. No differences between any individual pairs of groups or changes with time were observed (Figure 11C).

## Discussion

The overall aim of this work was to understand how traumatic SCI affects the N-glycosylation profile of the spinal cord, with a view to using this knowledge to develop and improve future biomaterial-based therapies for SCI. The rat is commonly employed in models of SCI, and the transection model used here is useful in studies of implantable biomaterials.^52^ This model was the primary focus to understand the mammalian glycosylation response to injury and how this may be influenced by a collagen hydrogel treatment. Modeling SCI in the frog *X. laevis* allowed us to study the differences in the glycosylation response in a permissive regeneration environment and compare that to an environment that is inhibitory to regeneration, which could help to identify potential glycosylation pathways that might correlate with loss of regeneration. Linking the glycosylation studies performed in *X. laevis* with those performed in the rat most notable differences in the response to SCI was in glycan branching and complexity. In the injured rat spinal cord, there was an overall decrease in complex glycans in favor of the oligomannose species and the hybrids (Figure 5). The observed increase in both GlcNAc and sialic acid suggests defined glycosylation signatures associated with the regenerative response following SCI, not just a general increase in complexity and elongation of glycan branches.

We first established the normal N-glycoprofile of the intact adult female rat spinal cord. Our findings broadly agree with the mass spectrometric glyco-profile recently described by Osimanjiang et al.^16^ Oligomannose, hybrid, and complex N-glycans were identified. Of the complex glycans, most were multiantennary, and core and outer fucose was typical, with a high level of galactosylation, including some α-galactose. However, our analysis identified bisecting GlcNAc residues, while we did not assign any poly-LacNAc structures. Additionally, we identified sulfated structures and acetylated sialic acid (Figure 4, Supporting Tables S6 and S7). Lectin histochemistry was used to study the glycosylation response in *X. laevis*. DSA lectin binds terminal GlcNAc, which is key to establishing the degree of branching of an N-glycan, as opposed to its being oligomannose type. High terminal GlcNAc also indicates a low level of branch extension with galactose. SNA-I lectin binds terminal sialic acid, which may be attached to galactose to terminate a branch of a complex N-glycan.

Injury to the spinal cord resulted in disruption of the N-glycoprofile, most noticeably in the lesion epicenter. An overall loss of complexity in N-glycan species was observed in response to the transection injury in rat (Figure 5) with only subtle differences in the presence of collagen hydrogel (Figure 6). The increase in oligomannose and nonfucosylated structures at the expense of complex glycans suggests either a failure of the N-glycosylation machinery in the Golgi apparatus^8^ in response to SCI or changes in the synaptic machinery.^53,54^ Increased bisecting GlcNAc has been observed in Alzheimer’s and Huntington’s disease^19,21,55^ and here may be a result of widespread cell death. Increased core fucosylation has also been observed in these neurodegenerative diseases,^21,55,56^ but this was not found to be a feature of SCI. Increased oligomannose and hybrid structures potentially exacerbated and sustained the inflammatory response after SCI in rat. Many immune cell receptors have mannose-binding activity for the recognition of pathogens.^57^ Normally macrophages are not exposed to the oligomannose structures typical of CNS glycans,^58^ but in SCI, the blood spinal cord barrier is disrupted, with an influx of peripheral macrophages^59^ bringing these cells into contact with the “apparently foreign” oligomannose glycans, and likely exacerbating the inflammatory response.^60^ In contrast, in *X. laevis*, there seemed to be an increase in branched complex glycans (increased DSA binding to GlcNAc) in the vicinity of the injury, particularly in the regenerating tadpole at the earlier time point, indicating more complexity in the glycans present (Figure 8). This suggests that a transient increase in N-glycan complexity may contribute to repair of the spinal cord.

This disruption of the blood spinal cord barrier may contribute to the observed increase in sialic acid in the lesion epicenter at 7 dpi (Supporting Figure S4) as serum proteins are quite highly sialylated.^58,61,62^ Increased sialylation was also seen using SNA-I lectin histochemistry in a rat contusion model.^17^ The collagen hydrogels had a hemostatic effect at the time of surgery (Supporting Figure S6), and this may contribute to the smaller sialylation response observed. Sialic acid is widely known to play a role in the regulation of the immune system, and inflammation^11,63^ and here double labeling with lectin- and immunohistochemistry showed CD11b-positive cells in close proximity to positive SNA-I staining (Figure 7). In *X. laevis*, there was an early increase in sialic acid following injury in tadpole and particularly in froglet (Figure 9). Considering the association of sialic acid with microglia/macrophages in rat, attempts were made to perform similar double-labeling experiments in the injured *X. laevis* spinal cord but satisfactory immunolabeling could not be achieved. However, double labeling with astrocytic and neuronal markers showed that these cells did not carry sialic acid (Supporting Figure S10). It seems worthwhile to investigate sialic acid as a tool to manipulate the inflammatory response post-SCI. For example, sialic-acid-binding siglecs and/or selectins, which are important in recruitment and extravasation of macrophages,^64,65^ and which have been observed to be differentially expressed in response to various traumatic or inflammatory central nervous system pathologies,^65−67^ could be targeted.

WFA was used to investigate GalNAc, which is a significant component of the GAG chains found on CSPGs. These GAG chains have been proposed to contribute some of the neurite inhibitory properties of the glial scar which forms after mammalian SCI.^68,69^ The result for GalNAc here was surprising as GalNAc (WFA staining) increased in the tadpole spinal cord after transection and less GalNAc was detected in the nonregenerating froglet spinal cord, decreasing further over time (Figure 10). This result contradicts the established idea of CSPG GAG chains inhibiting neuronal growth; however, it is possible that WFA has labeled *O*-linked GalNAc here. Extensive *O*-GalNAc was observed in the developing mouse nervous system^70^ and has been associated with tumor progression and metastasis.^71,72^ It is possible that increased WFA labeling here is related to cell division, migration, or axonal outgrowth.

**Figure 12:**
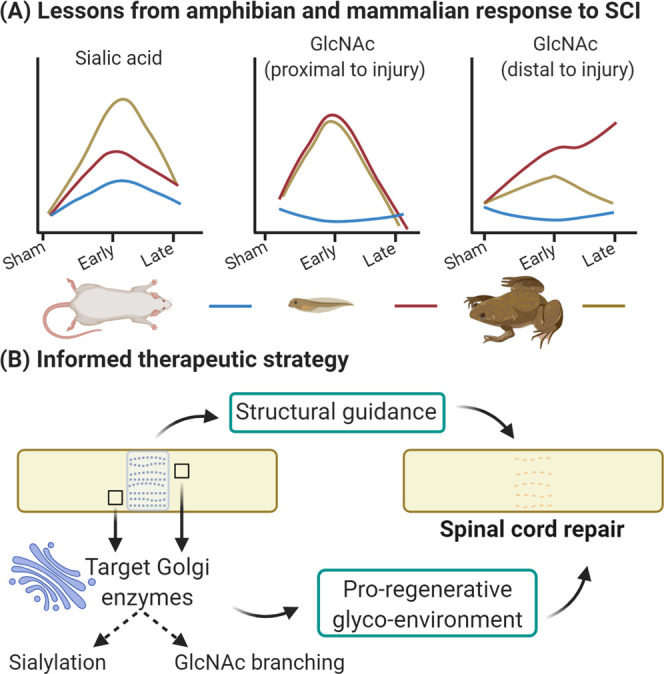
Summary and future perspectives. (A) Between rat and *Xenopus*, pre- and post-metamorphosis, there were distinct glycosylation responses to SCI. In the regenerative tadpole, there was a moderate increase in sialylation, a transient increase in GlcNAc in close proximity to the injury, and a continued increase in GlcNAc distant from the injury. (B) Glyco-enzymes of the Golgi could be targeted, ideally in a spatiotemporal manner, to create a more proregenerative environment in the injured spinal cord which, when combined with the structural cues and guidance provided by an aligned collagen hydrogel, should encourage repair of the injured spinal cord. Created with BioRender.com.

This work describes a comprehensive N-glycoprofile of the rat spinal cord and is the first report of the changes associated with transection injury and biomaterial treatment, specifically collagen hydrogel. In addition, we have examined some of the glycan features associated with regenerative success or failure using the *X. laevis* model. From this study, we can say that SCI disrupts the typical N-glycosylation profile of the rat spinal cord and that it is likely that specific glycosylation signatures support regeneration. In particular, increased GlcNAc branching may be important for the regenerative response (Figure 12A). The lack of effect on the glycoprofile seen with collagen hydrogel treatment reinforces the idea that a combinatorial treatment may be necessary. Specifically, the glyco-enzymes of the Golgi apparatus in the vicinity of the injury should be targeted to appropriately regulate sialylation and GlcNAc branching. Such strategies should encourage a more permissive glyco-environment and enhance the structural guidance and support provided by the aligned hydrogel (Figure 12B).

## Supporting Information Available

ROIs for image acquisition in *X. laevis* spinal cord tissue (Figure S1); linkage analysis chromatograms of spinal cord glycans; (Figure S2); changes in the N-glycoprofile in the lesion epicenter of the injured rat spinal cord (Figure S3); WAX-HPLC profile of undigested N-glycans from the lesion epicenter of the rat spinal cord (Figure S4); distribution of sialic acid labeled with SNA-I lectin and its relationship to astrocytes and neurons in the rat spinal cord (Figure S5); treatment with collagen hydrogel reduces bleeding at the site of injury (Figure S6); representative images of GlcNAc distribution in the spinal cord of pre- and postmetamorphic *X. laevis* (Figure S7); representative images of sialic acid distribution in the spinal cord of pre- and postmetamorphic *X. laevis* (Figure S8); representative images of GalNAc distribution in the spinal cord of pre- and postmetamorphic *X. laevis* (Figure S9); representative images of the lesion epicenter of injured tadpole spinal cord (Figure S10); solvent gradients, flow rate, and column temperature over each 30 min HILIC-UPLC run (Table S1); solvent gradients, flow rate, and column temperature for DMB analysis (Table S2); solvent gradients, flow rate, and column temperature over each 30 min run on LC-MS (Table S3); parameters for the mass spectrometer (Table S4); significant glycans of each peak determined and translation of these structures to the N-glycoprofile obtained in the SCI study (Table S5); and SCI and collagen hydrogel implantation in the rat, SCI in *X. laevis*, Lectin- and immunohistochemistry, and image acquisition and analysis (Supporting Methods) (PDF)
Glycan structures identified in each peak of each exoglycosidase digest (Table S6); undigested N-glycans analyzed using a Waters Xevo G2 Q-TOF mass spectrometer in negative mode (Table S7) (XLSX)
