# Supplementary material for: Distinct Glycosylation Responses to Spinal Cord Injury in Regenerative and Nonregenerative Models
Source: J Proteome Res. 2022 May 4;21(6):1449–66. doi: 10.1021/acs.jproteome.2c00043 (PMC9171824; doi:10.1021/acs.jproteome.2c00043)
Supplement: Supplementary file 1 — pr2c00043_si_001.pdf [file pr2c00043_si_001.pdf]

Distinct glycosylation responses to spinal cord injury in regenerative and non-regenerative models

Rachel Ronan[1,8], Aniket Kshirsagar[1], Ana Lúcia Rebelo[1], Abbah Sunny[1], Michelle Kilcoyne[2], Roisin O' Flaherty[3,4], Pauline M. Rudd[4,5], Gerhard Schlosser[6], Radka Saldova[1,4,7#], Abhay Pandit [1#], Siobhan McMahon[8#]*.

[1]SFI Research Centre for Medical Devices (CÚRAM), National University of Ireland Galway, Ireland, H91 W2TY; [2]Discipline of Microbiology, National University of Ireland Galway, Ireland, H91 W2TY; [3]Department of Chemistry, Maynooth University, Maynooth, Co., Kildare, Ireland, W23 F2H6; [4]The National Institute for Bioprocessing, Research, and Training (NIBRT), Dublin, Ireland A94 X099; [5]Conway Institute, University College Dublin, Belfield, Dublin 4, D04 PR94, [6]School of Natural Science, National University of Ireland Galway, Ireland, H91 W2TY; [7]UCD School of Medicine, College of Health and Agricultural Science (CHAS), University College Dublin (UCD), Dublin, Ireland; D04 PR94, [8]Discipline of Anatomy, National University of Ireland, Galway, Ireland, H91 W5P7.

*Dr Siobhan McMahon, Discipline of Anatomy, College of Medicine Nursing and Health Sciences, National University of Ireland Galway, Ireland. **Email:** siobhan.mcmahon@nuigalway.ie **Telephone:** +353 91492838

[#]SMcM, RF and AP should be considered joint senior author.

Table of contents:

Figure S1: ROIs for image acquisition in Xenopus laevis spinal cord tissue.
Figure S2: Linkage analysis chromatograms of spinal cord glycans.
Figure S3: Changes in the N-glycoprofile in the lesion epicentre of the injured rat spinal cord.
Figure S4: WAX-HPLC profile of undigested N-glycans from the lesion epicentre of the rat spinal cord.
Figure S5: Distribution of sialic acid labelled with SNA-I lectin and its relationship to astrocytes and neurons in the rat spinal cord.
Figure S6: Treatment with collagen hydrogel reduces bleeding at the site of injury.
Figure S7: Representative images of GlcNAc distribution in the spinal cord of pre- and post-metamorphic Xenopus laevis.
Figure S8: Representative images of sialic acid distribution in the spinal cord of pre- and post-metamorphic Xenopus laevis.
Figure S9: Representative images of GalNAc distribution in the spinal cord of pre- and post-metamorphic Xenopus laevis.
Figure S10: Representative images of the lesion epicentre of injured tadpole spinal cord.
Table S1: Solvent gradients, flow rate and column temperature over each 30 minute HILIC-UPLC run.
Table S2: Solvent gradients, flow rate and column temperature for DMB analysis.
Table S3: Solvent gradients, flow rate and column temperature over each 30 min run on LC-MS.
Table S4: Parameters for the mass spectrometer.
Table S5: The significant glycans of each peak determined and translation of these structures to the N-glycoprofile obtained in the SCI study.
Table S6: Glycan structures identified in each peak of each exoglycosidase digest.
Table S7: Undigested N-glycans analysed using a Waters Xevo G2 Q-TOF mass spectrometer in negative mode.
Supplementary Methods: SCI and collagen hydrogel implantation in the rat, SCI in Xenopus laevis, Lectin- and immunohistochemistry, Image acquisition and analysis

Supplementary Figures

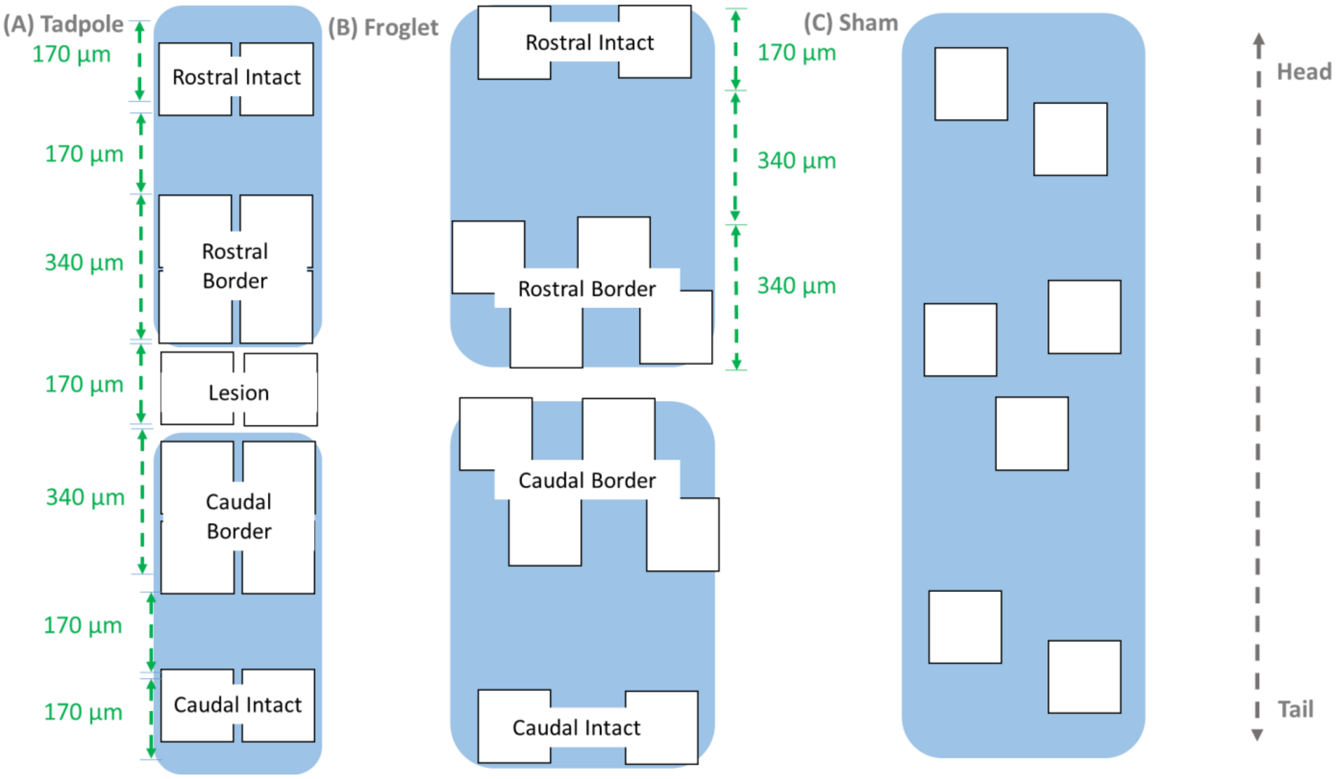

Supplementary Figure 1: ROIs for image acquisition in *Xenopus laevis* spinal cord tissue. Images were acquired from 5 ROIs for tadpole (A) and 4 ROIs for froglet (B). In both developmental stages, images for sham animals were acquired from an area at an equivalent level to that for injured animals (C).

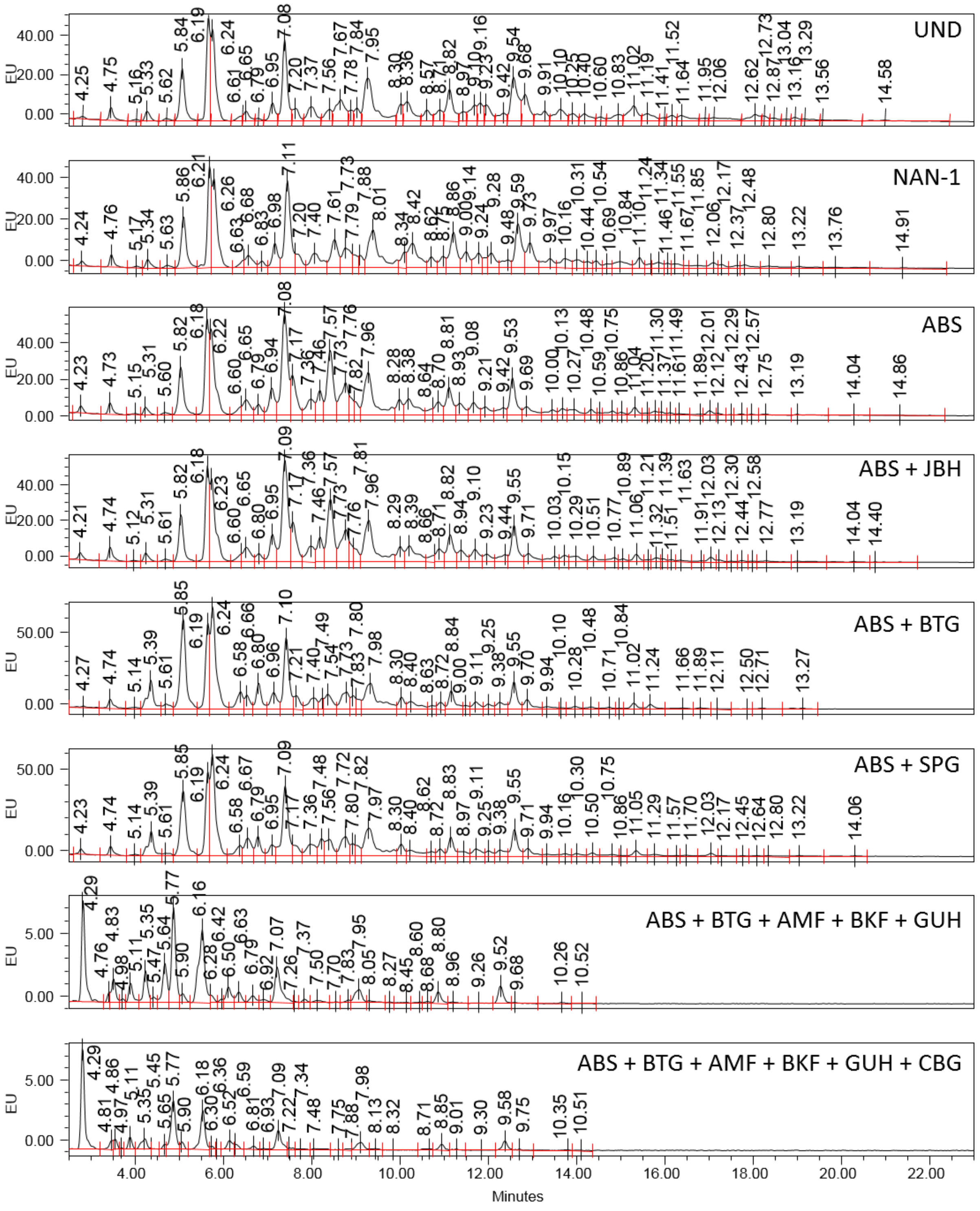

Supplementary Figure 2: Linkage analysis chromatograms of spinal cord glycans. NAN-1 removes α(2-3) linked sialic acids only, ABS removes α(2-3, 6, 8) linked sialic acids. JBH is a hexosaminidase which digests both GlcNAc and GalNAc. BTG removes β(1-3) and (1-4) linked galactose while SPG removes only β(1-4)-galactose. CBG removes α(1-3), and α(1-4) linked galactose.

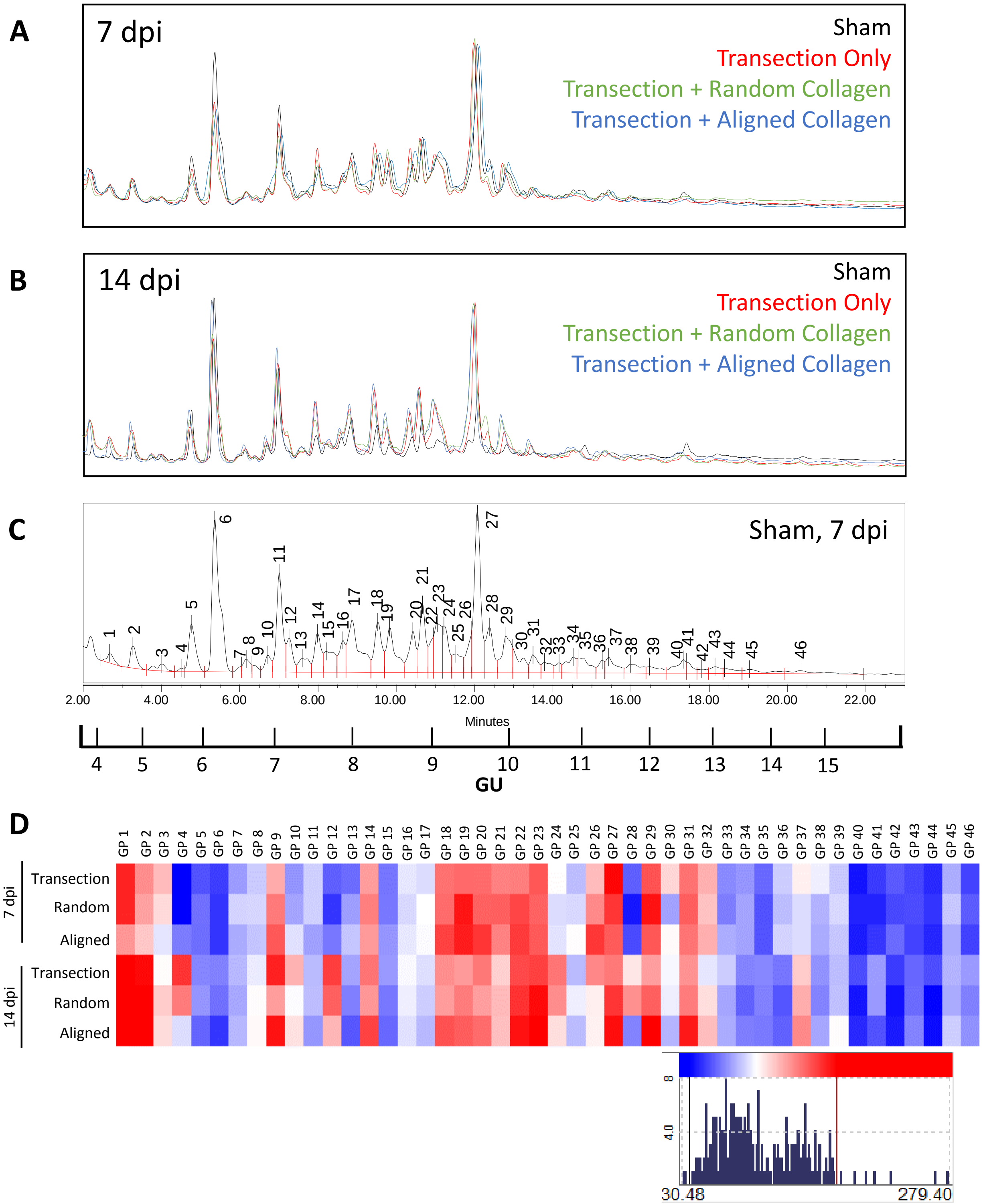

Supplementary Figure 3 Changes in the *N*-glycoprofile in the lesion epicentre of the injured rat spinal cord. (A) Representative profiles for 7 dpi groups, overlaid, (B) representative profiles for 14 dpi groups, overlaid, (C) example of integration, (D) heat map demonstrating the fold change from the sham group at 7 or 14 dpi. Peaks are labelled with the glycan peak (GP) number. GU, glucose units are shown as a scale.

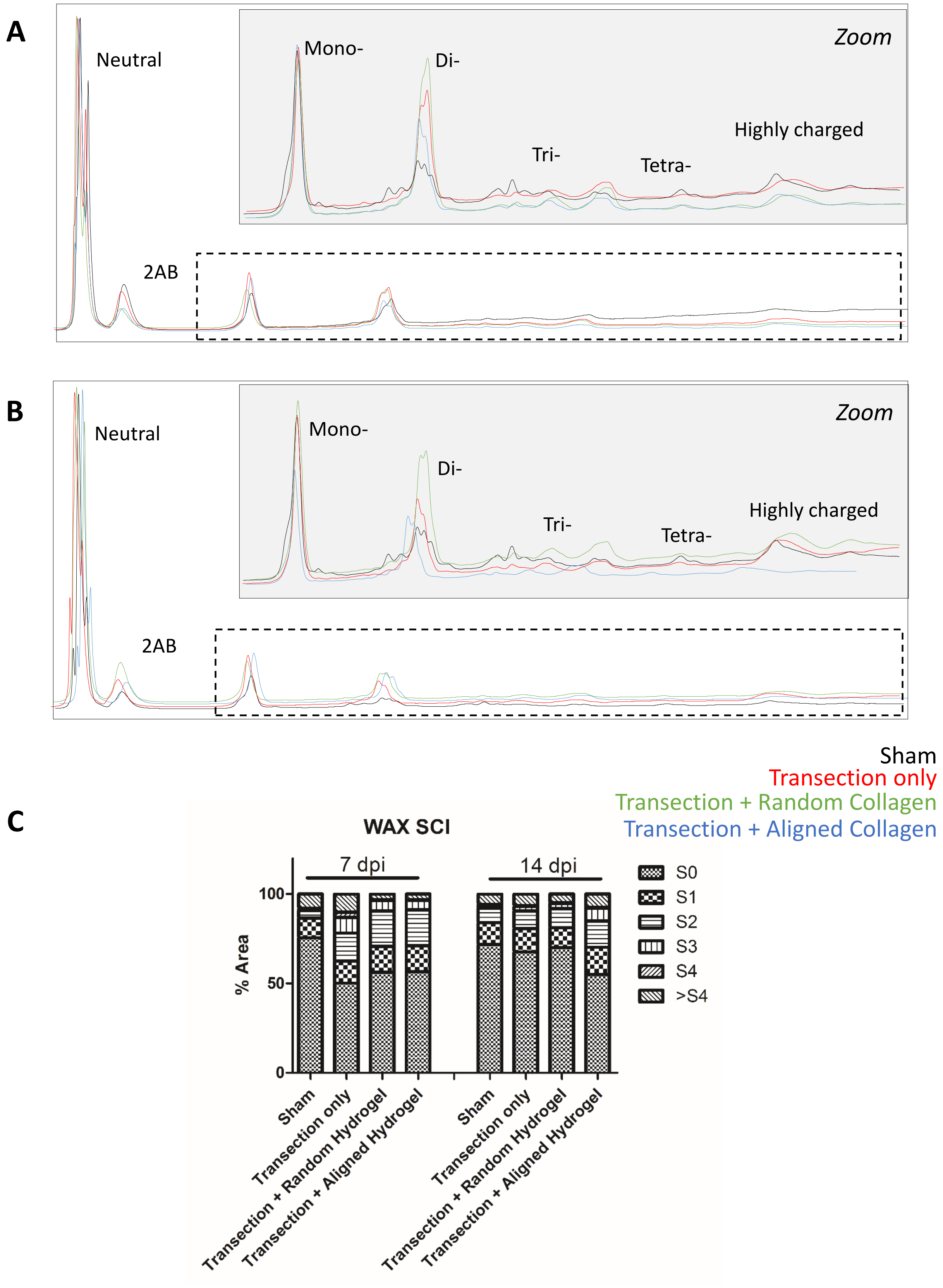

Supplementary Figure 4 WAX-HPLC profile of undigested N-glycans from the lesion epicentre of the rat spinal cord at (A) 7 and (B) 14 dpi. The charged region is shown highlighted in grey, inset into the full view chromatogram, with dashed line indicating the zoomed region.

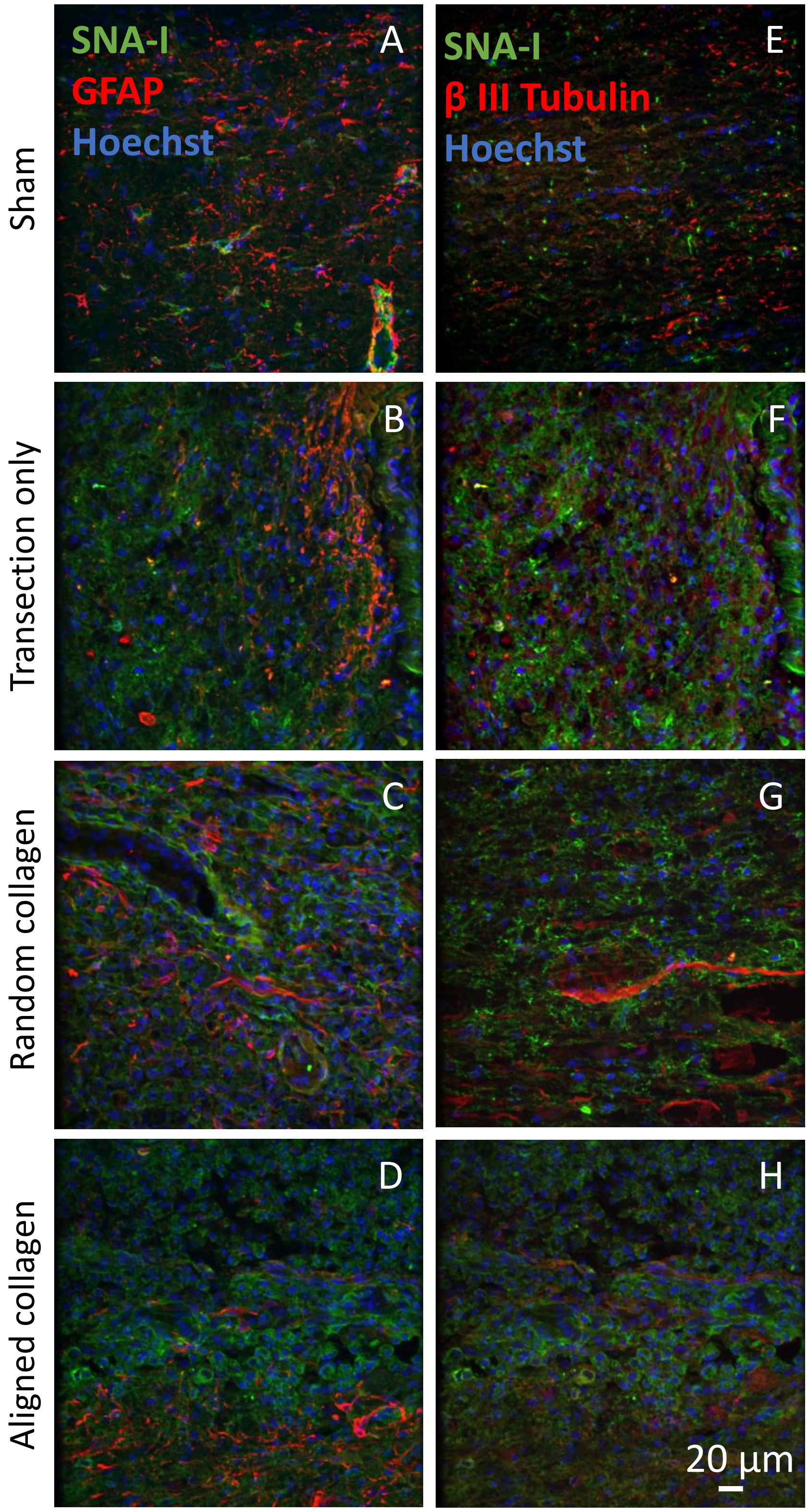

Supplementary Figure 5 Distribution of sialic acid labelled with SNA-I lectin and its relationship to astrocytes and neurons in the rat spinal cord. Images A-D show association of SNA-I lectin and GFAP positive astrocytes. Images (E-H) show association of SNA-I staining and βIII tubulin positive neurons. (A, E) Sham, (B, F) transection only, (C, G) random collagen hydrogel treated, (D, H) aligned collagen hydrogel treated. Images B-D and G-H were captured at the borders of the injury. No positively stained astrocytes or neurons were seen in the lesion epicentre. No co-localisation was observed between SNA-I and either marker. All images are from the spinal cord at 7 dpi. Scale bar 20 µm.

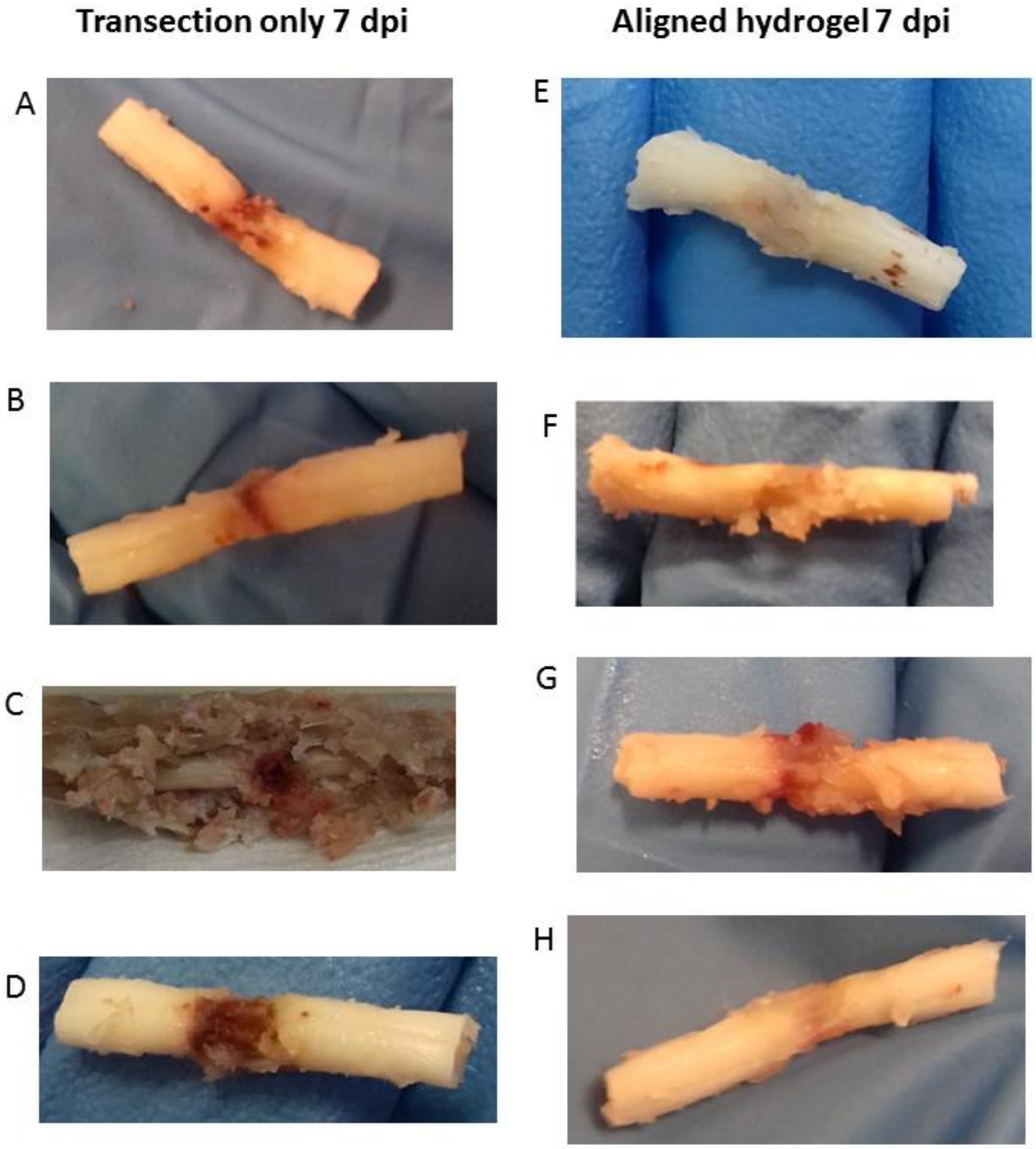

Supplementary Figure 6: Treatment with collagen hydrogel reduces bleeding at the site of injury. Images were captured at the time of tissue dissection, post fixation in 4% PFA. In the transection only group (A-D) there appeared to be a large blood clot at the site of injury. In those animals treated with aligned collagen hydrogel (E-H) there was much less evidence of bleeding. Dpi, days post injury.

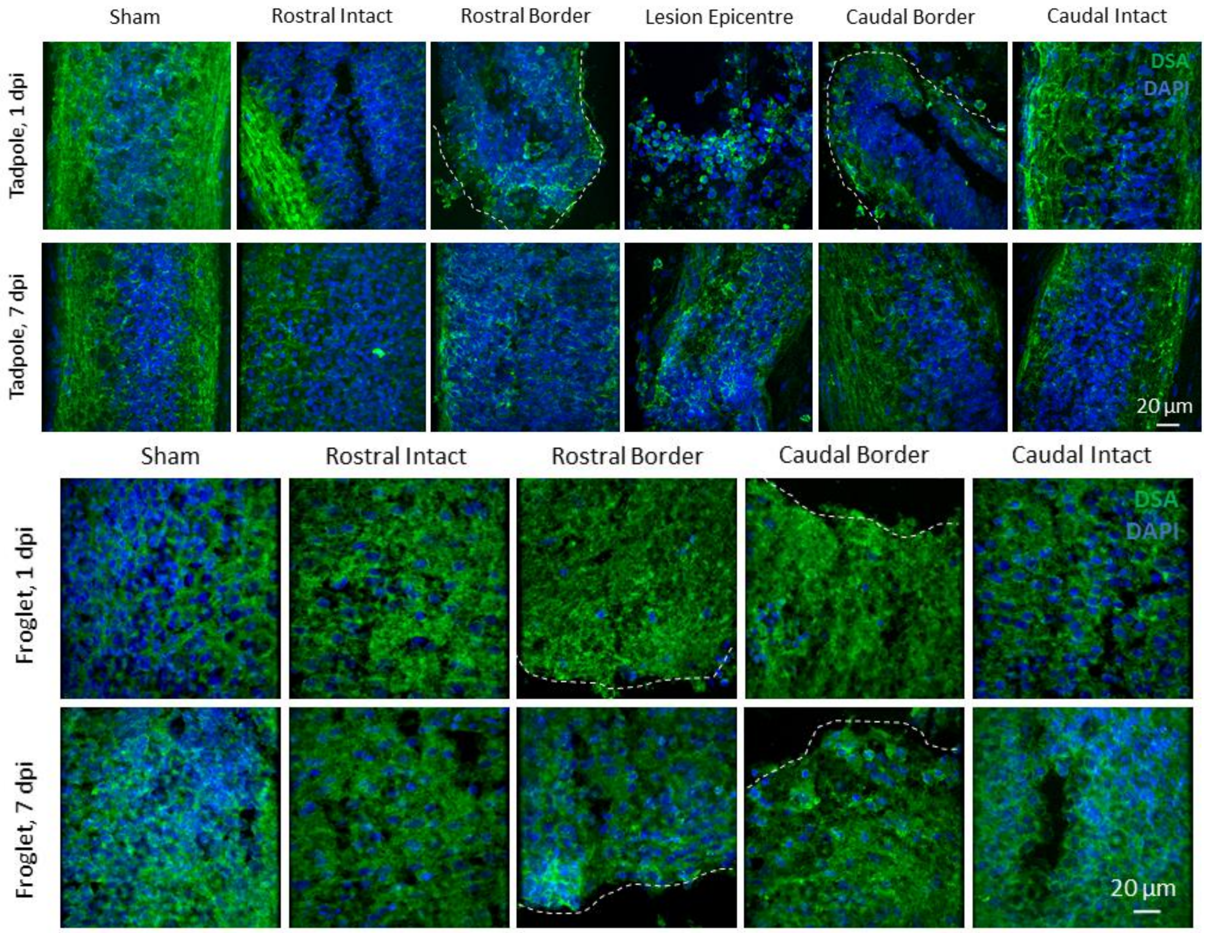

Supplementary Figure 7: Representative images of GlcNAc distribution in the spinal cord of pre- and post-metamorphic *Xenopus laevis* at 1 and 7 days following SCI. FITC-conjugated DSA lectin was used to label GlcNAc (green). Nuclei were labelled with Hoechst (blue). The edge of the lesion is outlined with a white dashed line.

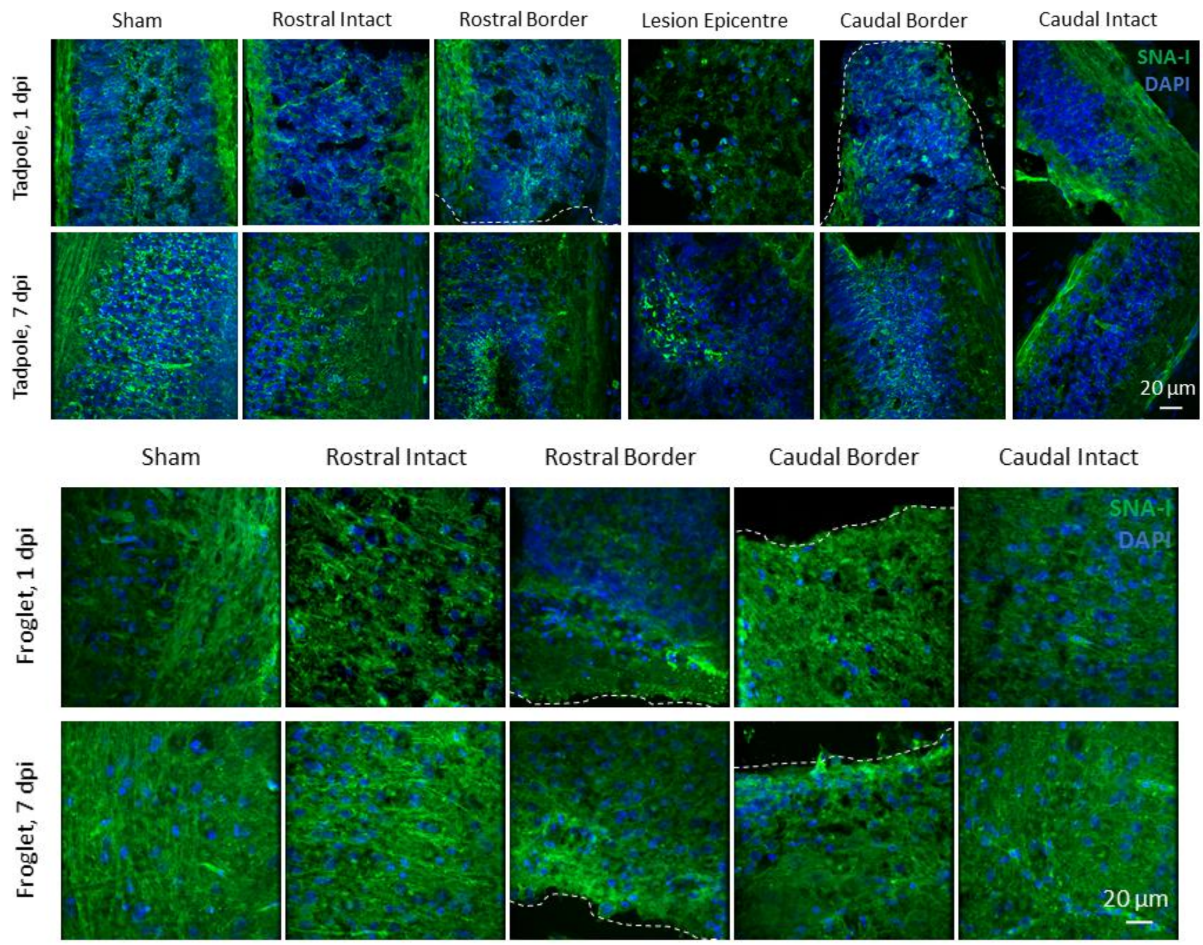

Supplementary Figure 8: Representative images of sialic acid distribution in the spinal cord of pre- and post-metamorphic *Xenopus laevis* at 1 and 7 days following SCI. FITC-conjugated SNA-I lectin was used to label sialic acid (green). Nuclei were labelled with Hoechst (blue). The edge of the lesion is outlined with a white dashed line.

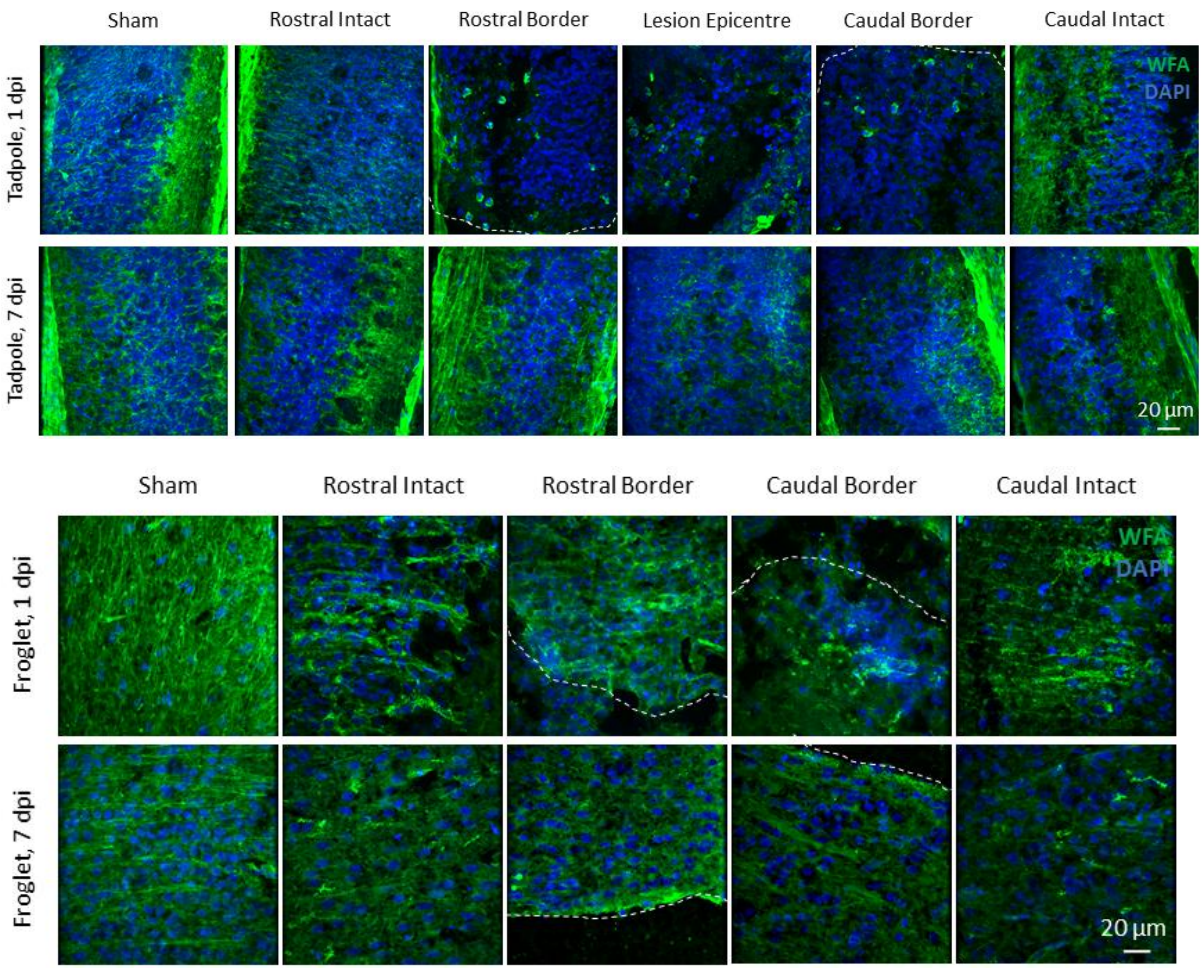

Supplementary Figure 9: Representative images of GalNAc distribution in the spinal cord of pre- and post-metamorphic *Xenopus laevis* at 1 and 7 days following SCI. FITC-conjugated WFA lectin was used to label GalNAc (green). Nuclei were labelled with Hoechst (blue). The edge of the lesion is outlined with a white dashed line.

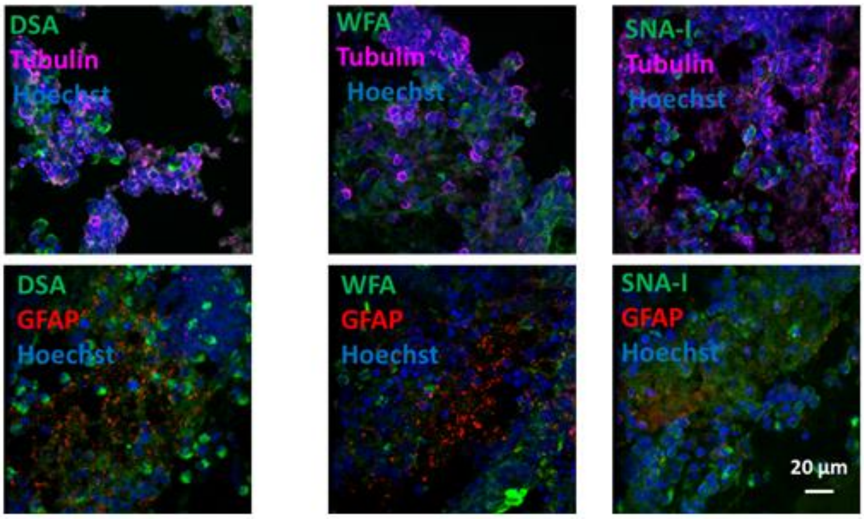

Supplementary Figure 10: Representative images of the lesion epicentre of injured tadpole spinal cord at 1 dpi. All lectins are FITC conjugated and shown in green, DSA labels GlcNAc, WFA labels GalNAc and SNA-I labels sialic acid. Tubulin was used as a neuronal marker and is shown in magenta, GFAP was used as an astrocytic marker and is shown in red. All nuclei were stained with Hoechst (blue). No association was seen between either marker with any of the three lectins studied, in any region of interest, in either tadpole or froglet tissue. Representative images from the tadpole lesion site at 1 dpi are shown here for illustration. Scale bar for all images = 20 µm.

Supplementary Tables

Supplementary Table 1: Solvent gradients, flow rate and column temperature over each 30 minute HILIC-UPLC run. Solvent A, 50 mM ammonium formate pH 4.4, Solvent B, 100% acetonitrile.

Time (mins)	% A	% B	Flow rate (ml/min)	Temperature (^{0}C)
0.00	30	70	0.561	40
24.81	47	53	0.561	40
25.50	70	30	0.561	40
26.25	70	30	0.300	40
26.55	30	70	0.300	40
28.50	30	70	0.400	40
30.00	30	70	0.561	40

Supplementary Table 2: Solvent gradients, flow rate and column temperature for DMB analysis. Solvent A, acetonitrile:methanol:water 9:7:84 (v/v); solvent B, acetonitrile.

Time (min)	Flow (mL/min)	%A	%B	Temperature (^{0}C)
0.0	0.25	100	0	30
7.0	0.25	100	0	30
7.5	0.25	10	90	30
8.0	0.25	10	90	30
8.5	0.25	100	0	30
15.0	0.25	100	0	30

Supplementary Table 3: Details of solvent gradients, flow rate and column temperature over each 30 min run on LC-MS. Solvent A, 50 mM ammonium formate pH 4.4; solvent B, 100% acetonitrile.

Time (min)	% A	% B	Flow rate (ml/min)	Temperature (^{0}C)
0.00	28	72	0.15	60
31.00	43	57	0.15	60
32.00	45	55	0.15	60
36.00	28	72	0.15	60
40.00	28	72	0.15	60

Supplementary Table 4: Parameters for the mass spectrometer.

Capillary voltage	1.8 kV
Sampling cone	50
Extraction cone	4
Source temperature	120 ^{0}C
Desolvation temperature	400 ^{0}C
Cone gas flow	40 L/hour
Desolvation gas flow	600 L/hour

Supplementary Table 5: The significant glycans of each peak determined in the characterisation study, and translation of these structures to the *N*-glycoprofile obtained in the SCI study. In the case where one glycan peak in the SCI study translates to multiple peaks from the characterisation study, the glycan with the highest overall abundance was chosen and is marked with an asterisk (*).The GU values shown for the SCI study are an average for the peak across all experimental animals. Dark blue squares, GlcNAc; green circles, mannose; yellow circles, galactose; red triangles, fucose; magenta diamonds, Neu5Ac type sialic acid; light blue diamonds, Neu5Gc type sialic acid. Unless otherwise stated, all galactose residues are in β(1-4) linkage, all outer arm fucose are in α(1-3) linkage, core fucose residues are in α(1-6) linkage, and the branching of the core pentasaccharide and antennae follow the conventional linkage rules. Bisecting GlcNAc is β(1-4) linked to the central mannose. G(*) indicates a galactose residue which has been modified with an unidentified chemical group causing the A1G(*)1Ga1 structure in the GUH digest to elute earlier than expected. For the complete list of all structures and how they digest with exoglycosidase enzymes, see Supplementary Table 6. For mass data for these structures, see Supplementary Table 7.

Glycan Peak (GU), Characterisation	Peak %Area, Characterisation	Glycan Peak (Average GU), SCI	Structure	
1 (4.25)	0.31	1 (4.25)	M3	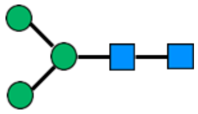
2 (4.75)	1.02	2 (4.72)	FM3	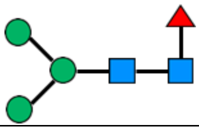
3 (5.16)	0.16	3 (5.26)	M4	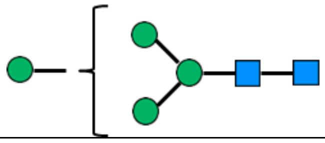
4 (5.33)	0.89		FA1*	* 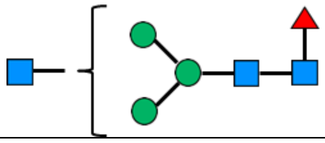

5 (5.62)	0.31	4 (5.63)	M4A1<br>FM4	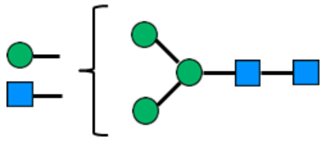<br>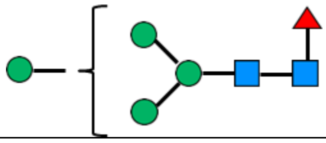
6 (5.84)	4.71	5 (5.80)	FA2	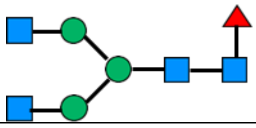
7 (6.19)	6.45		M5	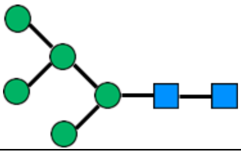
8 (6.24)	8.05	6 (6.17)	FA3*	* 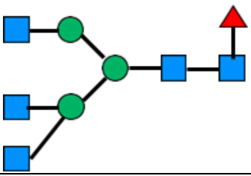
9 (6.61)	0.50	7 (6.57)	A4	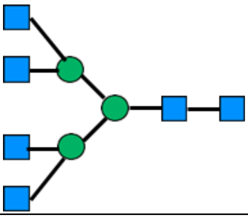
10 (6.65)	1.03	8 (6.63)	FM5	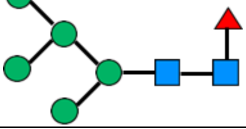
11 (6.79)	0.29	9 (6.77)	A2G2	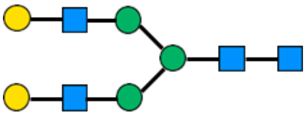
12 (6.95)	1.75	10 (6.94)	A3F(2)1G1S(2Ac)1	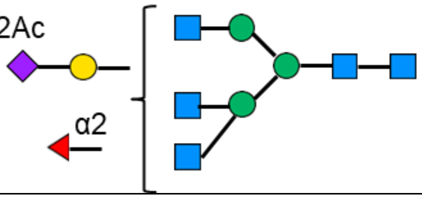
13 (7.08)	7.10	11 (7.08)	M6	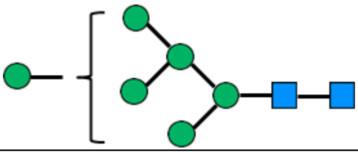

14 (7.20)	1.41	12 (7.19)	FA3G1	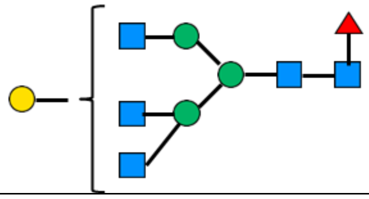
15 (7.37)	2.14	13 (7.38)	M6	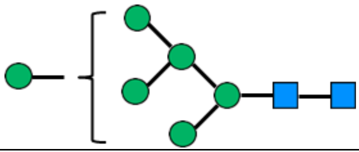
16 (7.56)	1.44	14 (7.55)	M6A1	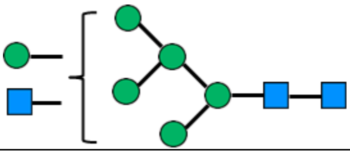
17 (7.67)	3.66	15 (7.69)	FM4A1F(2)1G1	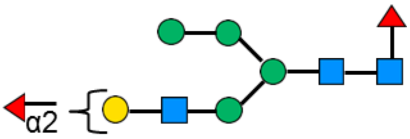
18 (7.78)	0.81		M7*	* 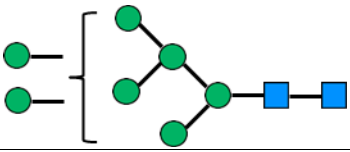
19 (7.84)	1.20	16 (7.85)	FA2F2G1 (both isomers with outer arm fucose F(2) and F(3) linked)<br>FA2F2G(SO_4^{2-})1	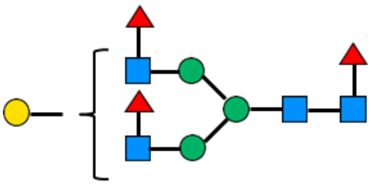<br>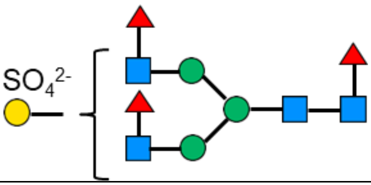
20 (7.95)	7.64	17 (7.97)	M7*	* 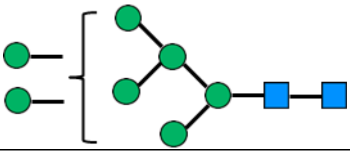

21 (8.30)	1.45		FA3F(2)3G1S(2Ac)1<br><br>A4G2	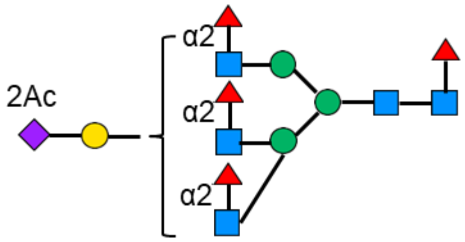<br>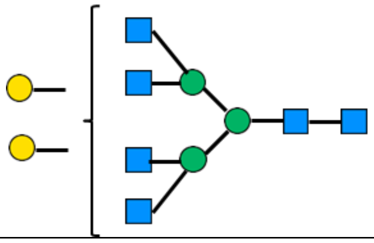
22 (8.36)	3.00	18 (8.27)	FA3G1S(6)1	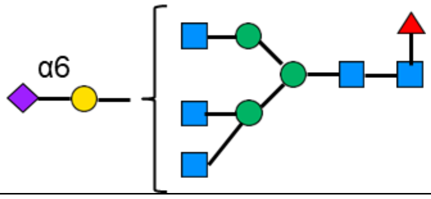
23 (8.57)	1.44	19 (8.42)	A3F2G(SO_4^{-2})1G1<br><br>FA3G1S(3)2	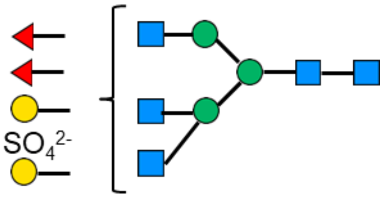<br>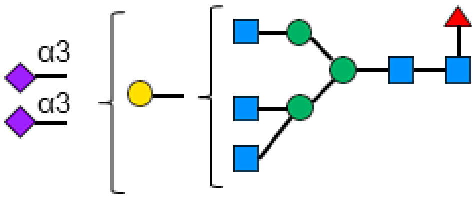
24 (8.71)	1.40	20 (8.70)	A3BG3	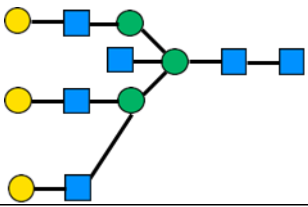
25 (8.82)	3.79	21 (8.82)	M8	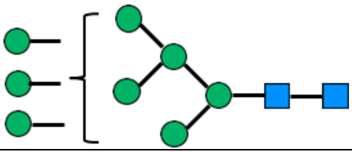

26 (8.97)	0.96	22 (8.95)	M8	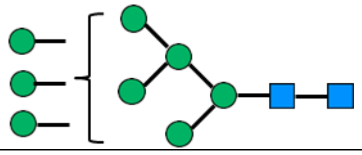
27 (9.10)	1.93	23 (9.02)	FA2G2Ga2	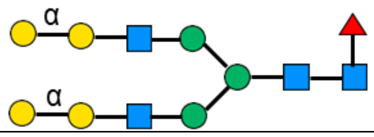
28 (9.16)	1.81	24 (9.09)	FA2G2S(3)2	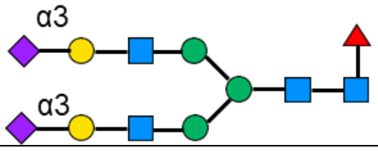
29 (9.23)	1.86	25 (9.26)	FA3F1G3S(2Ac)1<br>FM5A1G1S(6)1	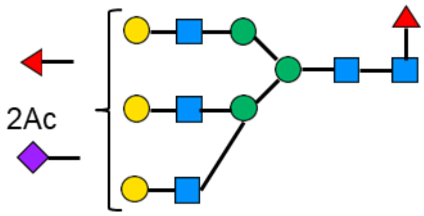<br>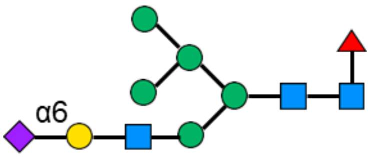
30 (9.42)	1.22	26 (9.46)	A2F(2)2G1Ga1S(6)1	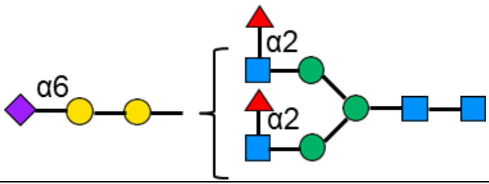
31 (9.54)	5.54	27 (9.54)	M9	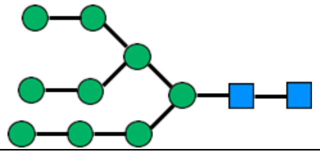
32 (9.68)	3.23	28 (9.71)	M9<br>A3G2S(6)2	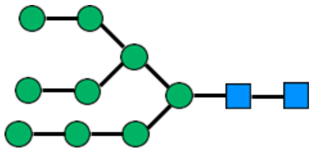<br>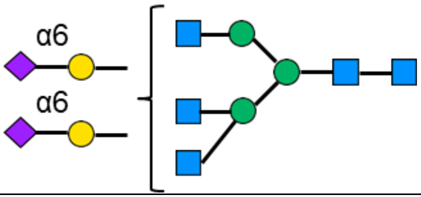

33 (9.91)	1.71	29 (9.93)	FA3F3G(SO$_4^{-2}$)1G1<br><br>A3F(2)1G(*)1G1Ga1	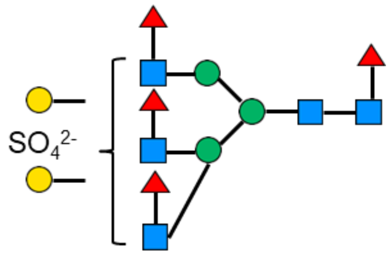<br>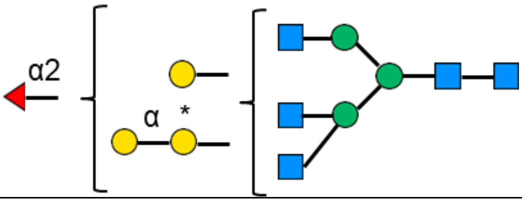
34 (10.10)	2.23	30 (10.10)	FA2G2Sg(3)1S(6)1	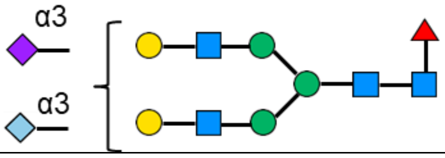
35 (10.25)	1.01	31 (10.32)	M9Glc1	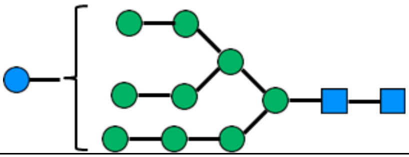
36 (10.40)	1.25	32 (10.54)	FA2F1G1Sg(6)1S(8)1<br><br>or<br><br>FA2F1G1S(6)1Sg(8)1<br><br>or<br><br>FA2F1G1Sg(6)1S(6)1	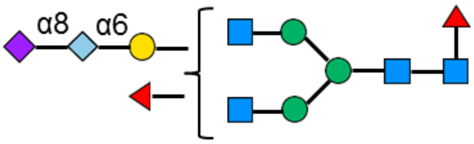<br>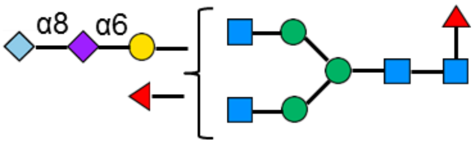<br>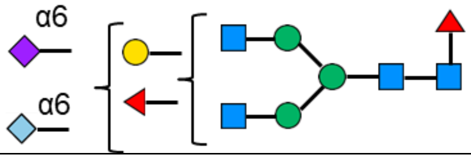
37 (10.60)	0.58	33 (10.71)	FA3F(2)3G3	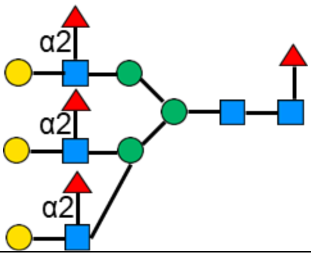

38 (10.83)	1.25	34 (10.91)	FA3G(SO_4^{-2})1G2Lac2<br>FA2F2G2S(6)2	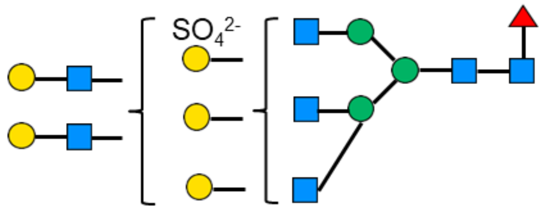 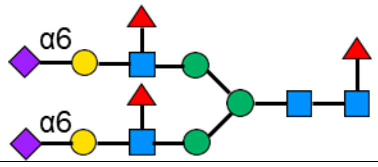
39 (11.02)	2.65	35 (11.03)	A2F(2)2G1Ga1Sg(3)2<br>FA3BF3G3<br>FA3F2G3S(2Ac)1	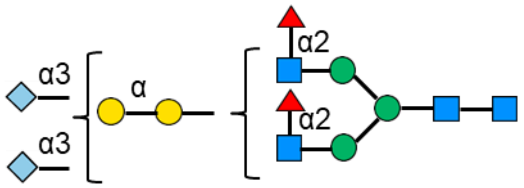 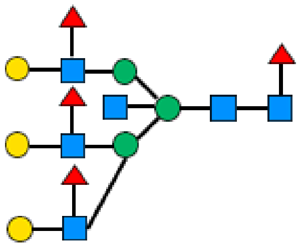<br>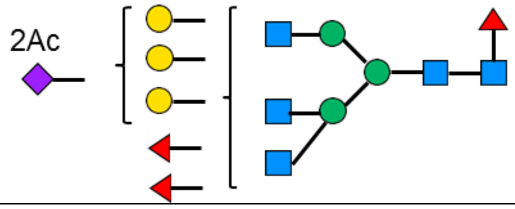
40 (11.19)	1.51	36 (11.34)	FA4F3G3	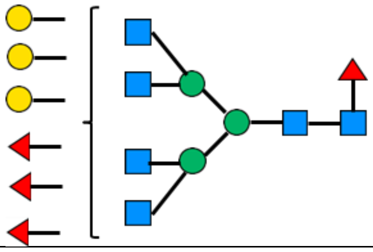
41 (11.41)	0.33	37 (11.44)	Sialylated A3 structures with or without fucose in core or outer arm positions	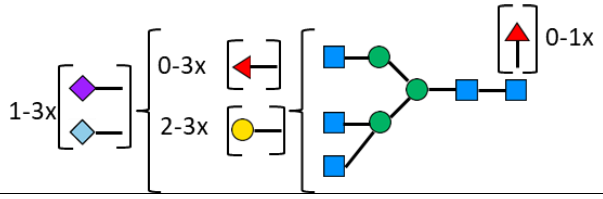

42 (11.52)	0.78		FA2G2S(6)2S(8)2<br>or<br>FA2G2S(6)4*	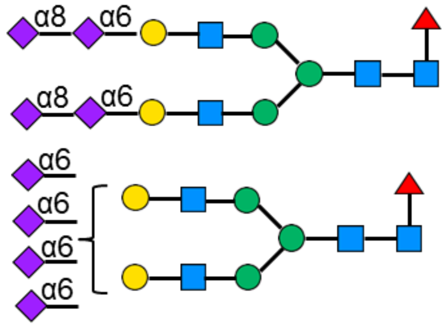<br>*
43 (11.64)	1.26	38 (11.80)	A3F(2)2F(3)1G1G(*)1Ga1	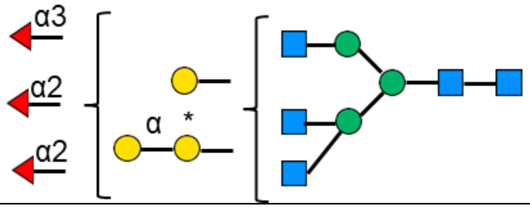
44 (11.95)	0.45		FA4F1G(SO$_4^{-2}$)1G2Lac2	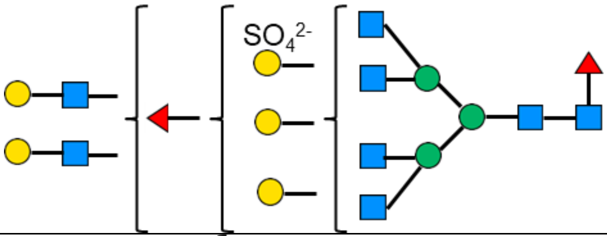
45 (12.06)	1.36	39 (12.03)	A4BG3Sg(3)1Sg(6)1<br>FA3F2G2Sg(6)1S(3)1<br>A4BF1G4Sg(3)1	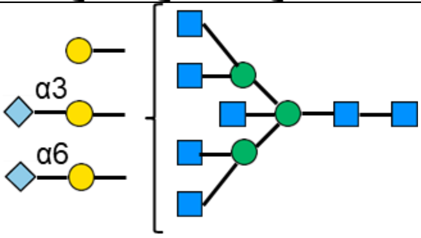<br>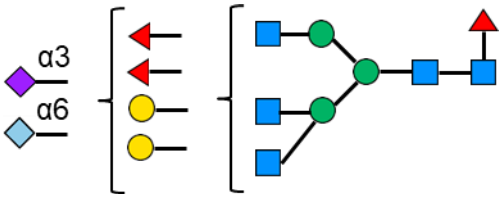<br>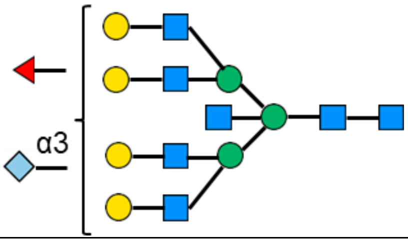

46 (12.62)	1.34	40 (12.55)	FA4F2G(SO_4^{-2})1G2Lac2	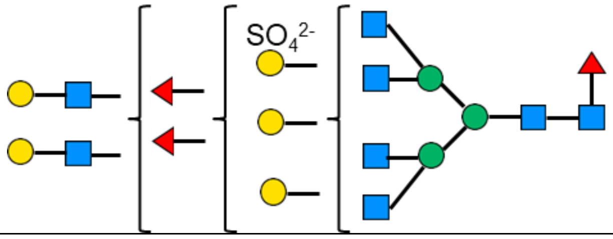
47 (12.73)	0.55	41 (12.61)	FA4F4G(SO_4^{-2}))1G2GlcNAc3	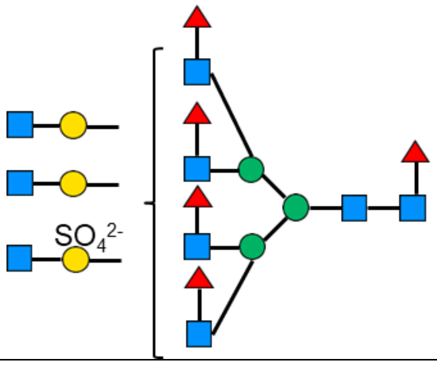
48 (12.87)	0.45	42 (12.85)	FA3F(2)3G1G(*)1Ga1Sg(3)1	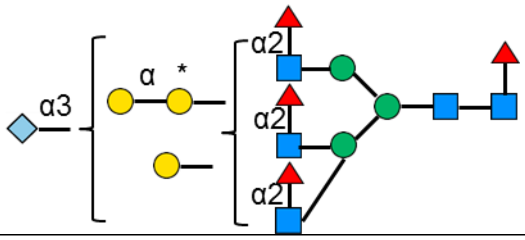
49 (13.04)	0.31	43 (13.03)	FA3F(2)3G1G(*)1Ga1Sg(3)1<br>(isomer of previous peak)	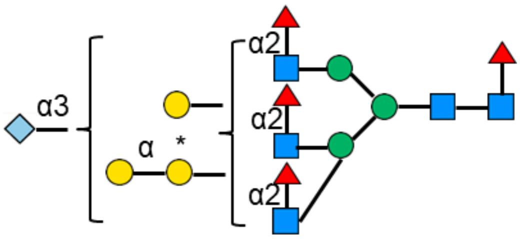
50 (13.16)	0.51	44 (13.19)	FA4F3G(SO_4^{-2})1G3GlcNAc4*	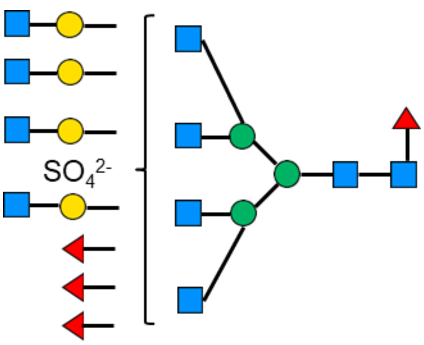<br>*

51 (13.29)	0.53		FA4F4G4S(6)1	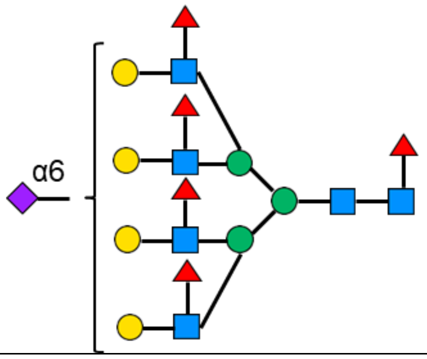
52 (13.56)	0.70	45 (13.59)	A3F(2)2G1G(*)1Ga1Sg(6)2	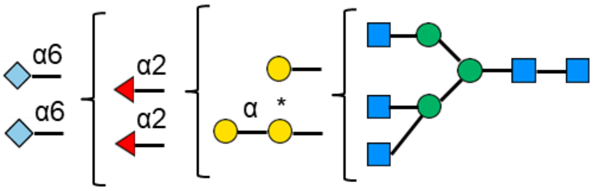
53 (14.58)	0.74	46 (14.48)	A4F1G4S(6)4	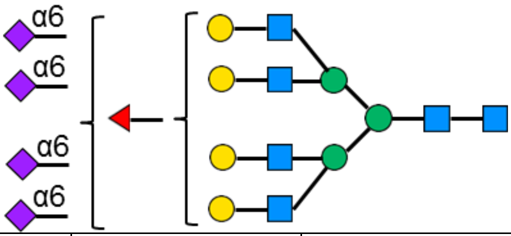

Monosaccharide symbols	GlcNAc	Mannose	Galactose	Fucose
	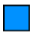	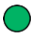	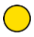	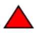
	Sialic acid (Neu5Ac)	Sialic acid (Neu5Gc)	Sulphate (on galactose)	Acetyl (on sialic acid)
	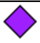	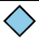	SO_4^{2-}	Ac
Conventional linkage	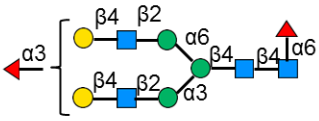		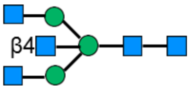	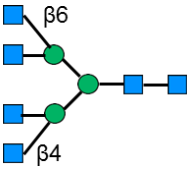

Supplementary Methods

SCI and collagen hydrogel implantation in the rat
Adult female Sprague-Dawley rats of weight 250 – 300 g (Charles River) were anaesthetized with isofluorane (Iso-vet, Chanelle group). The dorsum of the animal was shaved and cleaned with chlorhexidine (Hibiscrub). Surgery was performed on a heated table using aseptic technique.

A laminectomy at T9 was carried out to expose the spinal cord. At this stage, the wound was closed for animals designated as sham. To completely transect the spinal cord, a #15 scalpel (Swann-Morton) was passed through the spinal cord. For those animals receiving collagen hydrogel treatment, the hydrogel was trimmed to fit the wound and implanted immediately following transection of the cord into the resulting gap. Aligned hydrogels were implanted so that the fibers ran parallel to the direction of the cord. The wound was closed by suturing the muscle and skin in separate layers.

Post-operatively buprenorphine was administered for analgesia at 0.05 mg/kg; drinking water was supplemented with Dioralyte, animals were monitored twice daily for signs of infection, health and distress. Bladders were manually expressed twice daily. In the event of urinary infection, enrofloxacin was administered at 10 mg/kg.

At either 7 or 14 days post-injury (dpi) animals were euthanized by an overdose of sodium pentobarbitone. Transcardial perfusion was performed with 1X PBS. For those animals designated for glycomic analysis, the injured portion of the spinal cord was immediately dissected out, snap-frozen on dry ice and stored at -80 ^{0}C. Three regions of interest (ROIs) were collected: the lesion epicenter (10 mm of tissue centered on the lesion), and two 10 mm portions of tissue rostral or caudal to the injury. For animals designated for immunohistochemistry transcardial perfusion fixation with 4% paraformaldehyde (PFA) diluted in 1X PBS was performed. The injured segment of the spinal cord was removed and cryoprotected in 30% sucrose at 4 ^{0}C. Tissue was embedded in optimal cutting temperature (OCT) medium (Sakura), frozen in liquid nitrogen-cooled 2-methylbutane and cryosected at 20 µm thickness.

Collagen hydrogel preparation
Bovine type I collagen was dissolved in acetic acid at a concentration of 4 mg/ml and dialyzed. Aligned collagen fibers were formed by placing 300 µL collagen solution on a hydrophobic Teflon surface, and passing an electric current through for 15 minutes at 6 V. Collagen fibrils migrated to their isoelectric point, forming one continuous fiber of approximately 350 µm diameter. The collagen fiber was placed in phosphate buffered saline (PBS) overnight. Approximately 12-14 such fibers were arranged together to form the aligned collagen hydrogel for implantation into the transected rat spinal cord. The random collagen hydrogel was prepared from the same dialyzed solution. A drop of 1 mL was incubated overnight in PBS and was then ready for implantation into the spinal cord.

SCI in Xenopus laevis
Embryos were obtained following *in vitro* fertilization of oocytes and grown in-house until reaching Nieuwkoop and Faber (NF) stage 50 (pre-metamorphic tadpoles) or NF stage 66 (post-metamorphic froglets) (Nieuwkoop, Laboratory, & Faber, 1956). At all times, *Xenopus laevis* embryos were kept in 0.1X modified Barth solution (MBS). Animals were maintained at room temperature on a 12-hour light-dark cycle.

Stage 50 tadpoles were anaesthetized by immersion in 0.1% tricaine methanesulfonate (MS222, Fisher Scientific) diluted in 0.1X MBS for approximately 30 seconds or until loss of reflex responses. Excess MS222 was washed off in 0.1X MBS before immobilisation in plasticine. At the level of the sixth to the seventh myotome, the skin, muscle and dura were opened individually with fine forceps (Dumont #5, Fine Science Tools) to expose the spinal cord. The tip of a 25G needle (BD) was inserted ventrally to the spinal cord and pulled dorsally in one smooth movement, separating the spinal cord into two separate pieces. For sham injury, tadpoles were anaesthetized and immobilized in plasticine. Skin and muscle were opened at myotome six to seven as above, but spinal cord and dura mater were left intact.

Stage 66 froglets were anaesthetized by immersion in 0.5% MS222 for approximately 30 seconds or until loss of reflex responses. Excess MS222 was washed off in 0.1X MBS before immobilisation in plasticine. An incision through the skin and underlying connective tissue was made using a #11 scalpel (Swann Morton). A laminectomy was performed at the sixth vertebra using a Dumont #5 forceps (Fine Science Tools). The spinal cord was loosened from the surrounding vertebra and transected with fine scissors (Fine Science Tools). Complete transection was confirmed by passing the tip of the forceps

through the resulting gap in the tissue. To perform sham injury, froglets were anaesthetized and an incision made in the skin and connective tissue along the length of the spine as above. A laminectomy of the sixth vertebra was performed without touching the spinal cord.

Post-operatively both tadpoles and froglets were individually housed in 0.1X MBS supplemented with penicillin (10,000 units per ml), streptomycin (10 mg/ml) and gentamycin (2.5 mg/ml) (Sigma Aldrich) until the desired time-point. Feeding with a solution of finely ground nettle tea was re-introduced two days post-injury. Solutions were changed and health monitoring was carried out twice daily.

At either 1 or 7 dpi, euthanasia was carried out for both tadpoles and froglets by anesthetic overdose. Animals were immersed in 1% MS222 until cessation of heartbeat, and death was confirmed by the destruction of the brainstem region. Following euthanasia, the entire tadpole or the isolated vertebral column of froglets was fixed by immersion in 4% PFA in 1X PBS at 4 ^{0}C for 24 hours. Vertebral columns were cryoprotected in 30% sucrose. Tissue was placed in a small agarose mold surrounded by OCT and frozen in liquid nitrogen chilled 2-methylbutane. Tissue was stored at -80 ^{0}C and cryosectioned at 20 µm thickness.

Lectin- and immunohistochemistry

Rat or *Xenopus* tissue sections were brought to room temperature and washed in Tris buffered saline (TBS) containing 0.05% Triton-X-100 (TBS-T). Non-specific binding was blocked with a 2% solution of periodate treated bovine serum albumin (BSA) in TBS for one hour at room temperature. Following three washes in TBS-T, a solution of FITC-conjugated lectin in TBS-T was applied for sixty minutes at room temperature. Lectins in this study were purchased from EY labs and included SNA-I which binds terminal α(2-6) sialic acid (10 µg/ml), DSA which binds terminal *N*-acetyl glucosamine (GlcNAc, 5 µg/ml), and WFA which binds terminal *N*-acetyl galactosamine (GalNAc, 10 µg/ml). Sections were washed three times in TBS before incubating for 60 minutes at room temperature with primary antibodies (mouse anti-CD11b (EMD Millipore, 1:200), rabbit anti-GFAP (Dako, 1:400) and mouse anti-β-III Tubulin (Millipore, 1:200)) diluted in TBS-T. Sections were washed with TBS and incubated in secondary antibodies for 60 minutes at room temperature (donkey anti-mouse AF647 or donkey anti-rabbit AF594, Invitrogen) diluted 1:1000 in TBS-T. Secondary antibodies were washed off with TBS washes, counterstained with Hoechst (Invitrogen, 1:2000) for ten minutes before applying Fluoromount (Sigma Aldrich) and coverslips.

Image acquisition and analysis

All images were acquired with an Andor Revolution spinning disk confocal microscope (Andor Technology Ltd) at 40x magnification and viewed and analyzed in Fiji (version 1.50d, National Institute of Health, USA. Java 1.60_24 (64-bit)).

For *Xenopus* experiments, images were acquired from five ROIs in tadpoles and four ROIs in froglets. The lesion epicenter could not be stabilized during froglet tissue processing and was lost. Two to four images were acquired per ROI (Supplementary Figure 1).

Image analysis was performed in Fiji, employing a stereological approach together with Fiji's threshold and integrated density functions. Integrated density was chosen to incorporate both area and intensity of staining. A uniform threshold was used for all images for any given lectin. One optical slice was analyzed per image. The average per animal for each region was used for statistical analysis.

References

Nieuwkoop, P., Laboratory, H., & Faber, J. (1956). *Normal Table of Xenopus Laevis-Daudin. A Systematical and Chronological Survey of the Development from the Fertilized Egg Till the*

End of Metamorphosis. Edited by PD Nieuwkoop and J. Faber. Issued by the Hubrecht Laboratory, Utrecht.[With a Bibliography.]: North-Holland Publishing Company.
Royle, L., Campbell, M. P., Radcliffe, C. M., White, D. M., Harvey, D. J., Abrahams, J. L., . . . Dwek, R. A. (2008). HPLC-based analysis of serum N-glycans on a 96-well plate platform with dedicated database software. *Anal Biochem, 376*(1), 1-12. doi:10.1016/j.ab.2007.12.012
Samal, J., Saldova, R., Rudd, P. M., Pandit, A., & O'Flaherty, R. (2020). Region-Specific Characterization of N-Glycans in the Striatum and Substantia Nigra of an Adult Rodent Brain. *Anal Chem*. doi:10.1021/acs.analchem.0c01206
